# QSPR graph model to explore physicochemical properties of potential antiviral drugs of dengue disease through novel coloring-based topological indices

**DOI:** 10.3389/fchem.2025.1599715

**Published:** 2025-08-18

**Authors:** C. Yogalakshmi, B. J. Balamurugan

**Affiliations:** Department of Mathematics, School of Advanced Sciences, Vellore Institute of Technology, Chennai, Tamil Nadu, India

**Keywords:** dengue, isomorphic molecular graph, topological indices, color sum, physicochemical properties, QSPR analysis

## Abstract

Dengue is a viral disease transmitted to humans through mosquito bites. Researchers have investigated various drugs with potential antiviral properties against it. Some of the promising antiviral drugs include UV-4B (N-9-methoxynonyl-1-deoxynojirimycin), Lycorine, ST-148, 4-HPR, Silymarin, Baicalein, Quercetin, Naringenin, Nelfinavir, Ivermectin, Mosnodenvir (JNJ-1802), NITD-688, Metoclopramide, JNJ-A07 and Betulinic acid. The chemical structure of a drug can be modelled as an isomorphic molecular graph G(V,E) , considering the atoms as the vertex set V(G) and the bonds between the pair of atoms as the edge set E(G) . Graph coloring and topological indices serve as a powerful tools for analyzing the isomorphic molecular graph, providing the structural characterization and computational studies. In this article, two types of coloring-based topological indices *viz.*, chromatic topological indices and induced color-based topological indices, are introduced. Linear regression is employed in the QSPR(Quantitative Structure Property Relationship) analysis to examine the dengue antivirals through the computed topological indices of the aforementioned drugs. The results of the QSPR analysis reveal that the induced color-based indices provide better predictions of the physicochemical properties of dengue-treating drugs.

## 1 Introduction

An infectious virus called dengue infects people through the bite of infected mosquito species called Aedes. Tropical and subtropical regions are particularly at risk for dengue fever, which poses a significant public health threat. The signs and symptoms of dengue include high fever, severe headache, joint and muscle pain, rash, mild bleeding, pain behind the eyes, nausea, vomiting and mild respiratory problems. In severe cases, the symptoms may worsen and the individual may experience intense abdominal pain, persistent vomiting, rapid breathing, lethargy, restlessness, bleeding from the nose or gums, blood in vomit or stools and even organ failure. It also lead to drop in platelet count, which increases the risk of bleeding. It is hard to diagnose dengue fever since the symptoms of dengue is similar to many viral infections. Hence it is advised to run laboratory tests, such as reverse transcription-polymerase chain reaction (RT-PCR) or serological tests to diagnose and differentiate dengue from other infections. Numerous drugs have been evaluated through *in vitro*, *in vivo* and clinical studies to identify potential antivirals for dengue. In addition to these efforts, Dengvaxia, the first licensed dengue vaccine, has been developed to provide partial protection against dengue virus, although it is not a therapeutic antiviral. Furthermore, investigational drugs like JNJ-A07 and JNJ-1802, that target the dengue virus non-structural protein 4B (NS4B), have shown promising results in pre-clinical and clinical studies, demonstrating potent pan-serotype activity and potential to inhibit viral replication effectively. Several drugs have shown potential antiviral activity against the dengue virus, including UV-4B (N-9-methoxynonyl-1-deoxynojirimycin), Lycorine, ST-148, 4-HPR, Silymarin, Baicalein, Quercetin, Naringenin, Nelfinavir, Ivermectin, Mosnodenvir (JNJ-1802), NITD-688, Metoclopramide, JNJ-A07, and Betulinic acid. The targets and mechanisms of these drugs in combating the dengue virus are summarized in Table 1.

**TABLE 1:** The target and mechanism of the considered potential antiviral drugs of dengue.

S.No	Drug	Target	Mechanism	Reference
1	UV-4B	Endoplasmic reticulum-resident α -glucosidase 1 and α -glucosidase 2 enzymes	Inhibition of these enzymes prevents glycan processing and folding of viral glycoproteins, disrupting virus assembly, secretion, and fitness of nascent virions	Callahan et al. (2022)
2	Lycorine	RNA-dependent RNA polymerase (RdRp)	Lycorine binds at the palm and finger domains near the catalytic site of RdRp, interfering with negative strand viral RNA synthesis and disrupting viral replication	Agrawal et al. (2024)
3	ST-148	Capsid (C) protein	Inhibits the viral replication by targeting the capsid protein. Disrupts the virus’s ability to assemble and package its genome, preventing the formation of new viral particles	Touret et al. (2019)
4	4-HPR	Host lipid metabolism	Disrupts host lipid metabolism essential for viral replication, impairing the dengue virus life cycle	Martin et al. (2023)
5	Silymarin	Viral envelope(E) protein	Binds to the E protein with a binding affinity of −8.5 kcal/mol, forming hydrogen bonds with GLN120, TRP229, ASN89, and THR223	Low et al. (2021)
		Host cell membrane	Reduces viral entry efficiency into host cells (72.46 % ), impairing infectivity	
6	Baicalein	Host cell surface receptors	Blocks DENV attachment to Vero cells (95.59 % ), inhibiting the initial step of the viral life cycle	Low et al. (2021)
		Intracellular DENV replication machinery	Reduces production of DENV-3 intracellular progeny, disrupting replication and assembly processes	
7	Quercetin	Dengue virus envelope (E) protein	Binds to the E protein, interfering with viral entry into host cells by blocking the attachment and fusion processes	Singh et al. (2023)
8	Naringenin	Viral replication proteins and replication complex	Naringenin interferes with dengue virus (DENV) replication and/or maturation by targeting non-structural proteins essential for RNA replication and translation. It impairs the replication complex and shows effectiveness during and after the infection phase, reducing viral titers and replication efficiency	Frabasile et al. (2017)
9	Nelfinavir	proteases NS2B-NS3	Inhibits viral replication by targeting and blocking the NS2B-NS3 protease in DENV.	Bhakat et al. (2015)
10	Ivermectin	Dengue virus (DENV) replication	Inhibits viral replication by targeting specific components or processes necessary for the virus replication cycle	Suputtamongkol et al. (2021)
11	Mosnodenvir	NS4B	Inhibits dengue virus replication by targeting a viral protein (likely NS4B)	Bouzidi et al. (2024)
12	NITD-688	Nonstructural protein 4B (NS4B)	Inhibits DENV replication by binding to NS4B with high affinity across all serotypes, disrupting the NS4B/NS3 interaction. This prevents the formation of new NS4B/NS3 complexes and disrupts pre-existing complexes, ultimately inhibiting viral replication	?
13	Metoclopramide	Dopamine 2 Receptor (D2R)	Inhibits DENV infection by targeting D2R on host cells, which are positively associated with DENV infection. Metoclopramide acts as a D2R antagonist, blocking DENV binding and reducing DENV replication and neuronal cell cytotoxicity. This leads to antiviral effects both *in vitro* (reduced viral replication) and *in vivo* (reduced DENV-induced CNS neuropathy and mortality)	Shen et al. (2021)
14	JNJ-A07	Dengue virus non-structural protein 4B (NS4B) and NS4A-2K-NS4B precursor	Inhibits the interaction between NS2B/NS3 protease/helicase complex and NS4A-2K-NS4B cleavage intermediate, blocking the formation of vesicle packets (VPs) involved in DENV RNA replication. It prevents the *de novo* formation of VPs, disrupting the viral replication process early in the cycle	Kiemel et al. (2024)
15	Betulinic acid	Dengue virus non-structural protein 4B (NS4B)	Betulinic acid binds to NS4B with a binding energy of − 7.02 kcal/mol, suggesting its potential as an antiviral. This interaction may inhibit viral replication and reduce dengue virus pathogenesis	Ali et al. (2024)

Chemical graph theory (Wagner and Wang, 2018) is a part of graph theory which combines the principles of chemistry and graph theory. In chemical graph theory, the molecular structure of a chemical compound can be modelled in terms of an isomorphic molecular graph G(V,E) with atoms as vertex set V(G) and the bonds between the atoms as an edge set E(G) . The degree of an atom(vertex) v , denoted by d(v) , is the number of bonds(edges) incident to the atom v and the neighborhood of an atom v , denoted by N(v) , is the set of all atoms that are adjacent to v .

Topological index of a molecular structure is a numerical value computed based on the structure of a molecule graph. It converts the qualitative or abstract information of a molecule into a quantitative form. Various types of topological indices have been developed based on the different parameters of the molecular graph structures. These include distance-based indices, degree-based indices, neighborhood-based indices and connectivity-based indices. The Quantitative Structure-Property Relationship (QSPR) analysis of a molecular graph is carried out through the topological indices to establish mathematical relationships between the structural features of chemical compounds and their physical or chemical properties.

In recent studies, researchers have employed the Quantitative Structure-Property Relationship (QSPR) analysis using various topological indices to predict the physicochemical and ADMET (Absorption, Distribution, Metabolism, Excretion, and Toxicity) properties of diverse drug compounds. Among the various topological index variants, degree-based and neighborhood degree-based indices have been widely employed to evaluate their predictive capabilities for drug-like compounds. For instance, Tamilarasi and Balamurugan (2025) utilized these indices to predict the properties of antifungal drugs, while Arockiaraj et al. (2025) applied them to analyze compounds used in the treatment of lung cancer. Similarly, degree-based indices have been employed in the QSPR modeling of drugs targeting heart disease Kuriachan and Parthiban (2025); Hasani and Ghods (2024), blood cancer Zaman et al. (2024) and tuberculosis Abubakar et al. (2024). Their application extends to respiratory diseases as well, with studies exploring treatments for asthma Balasubramaniyan and Chidambaram (2023) and COVID-19 Ugasini Preetha et al. (2024); Das et al. (2023). It is noteworthy that these degree-based indices have been widely applied across various diseases, highlighting their utility and predictive power. In addition to degree-based indices, distance-based topological indices have also proven effective. For instance, Sardar and Hakami (2024) employed the distance-based indices to predict properties of drugs used in Alzheimer’s disease, while Huang et al. (2023) focused on anticancer agents. Recent literature also highlights the use of more specialized topological variants. Density-based indices were applied to study monkeypox-related drugs Kalaimathi and Balamurugan (2023), while reverse-sum Revan indices found use in analyzing antifiloviral drugs Tamilarasi and Balamurugan (2022). Thilsath parveen and Siddiqui (2024) explored domination distance-based indices and Shi et al. (2025) investigated temperature-based indices to model the properties of anticancer compounds.

In graph theory (Bondy and Murty, 2008), a graph coloring of the graph G(V,E) is an assignment of colors to the elements of the graph such as vertices or edges or both. The coloring of each element of the graph holds significance in its own distinct manner. In this article, the vertex coloring of a graph is considered. The vertex coloring c:V(G)→N , where N is the set of natural numbers, is said to be proper if no two adjacent vertices have the same color. Here, the set of natural numbers represents the set of colors. The minimum number of colors used to color the vertices of the graph is called as chromatic number and it is denoted as χ(G) . The graph coloring helps to study the structural properties of graphs by analysing the relationship between the number of colors used to color the graphs and various graph parameters such as vertex degree, connectivity, independent number, neighborhood set and more.

The graph coloring finds various applications in chemistry, particularly in representing molecular structures. The assignment of colors to the vertices helps in differentiating the types of atoms or functional groups within a molecule (Huckvale et al., 2023; Jin et al., 2020). In reaction network analysis, vertices are represented as chemicals and the two vertices are connected by an edge if the two chemicals are reactive with each other. Coloring these vertices facilitates the separation of reactive chemicals, aiding the chemical manufacturing industry in efficiently (optimally) storing non-reactive chemicals together in their warehouses. The minimum number of colors used determines the minimum number of compartments or rooms required for storing the chemicals. Graph coloring is also applicable in conformational analysis, enabling the identification of structural similarities or differences. Different isomers can be colored distinctly, contributing to the systematic exploration of atom alignments in a molecular structure.

The topological indices based on graph coloring can provide a comprehensive understanding of the molecular graphs, facilitating the prediction of the physical and chemical properties of the molecules. Therefore, the chromatic topological indices emerges in the field of chemical graph theory. Unlike traditional indices, these coloring-based indices provide an alternate method for analyzing molecular structures, to understand how the arrangement of colors influences the molecular properties.

The notion of chromatic topological indices was introduced by Johan kok et al. in (Kok et al., 2016). For any color set, $C=\left\{c_{1},c_{2},\ldots ,c_{l}\right\}$ and the coloring $\phi_{t}\left(G\right)$ for 1≤t≤l! of a graph G , Johan kok et al. (Kok et al., 2016) introduced the indices *viz.*, first chromatic Zagreb index $M_{1}^{\phi_{t}}\left(G\right)$ , second chromatic Zagreb index $M_{2}^{\phi_{t}}\left(G\right)$ and third chromatic Zagreb index $M_{3}^{\phi_{t}}\left(G\right)$ . The arrangement of colors in a graph is categorized as $\phi^{-}$ and $\phi^{+}$ coloring. In $\phi^{-}$ coloring, colors are assigned to the vertices in increasing order, maximizing the usage of each color before proceeding to the next color. In $\phi^{+}$ coloring, colors are assigned to the vertices in decreasing order, maximizing the usage of each color before proceeding to the next color.

In (Albina and Manonmani (2022); (2021); (Kok et al. (2017); Rose and Naduvath (2018)) the chromatic topological indices of some classes of graphs were determined. Following this, Smitha) Rose and Sudev Naduvath introduced several variants of chromatic indices *viz.*, chromatic total irregularity index (Rose and Naduvath, 2020a), injective chromatic zagreb indices (Rose and Naduvath, 2019), injective chromatic total irregularity index (Rose and Naduvath, 2019), equitable chromatic zagreb indices (Rose and Naduvath, 2020b) and equitable chromatic irregularity index (Rose and Naduvath, 2020b). In (Rose and Naduvath, 2019) and (Rose and Naduvath, 2020b), they computed the injective and equitabe chromatic Zageb indices and injective and equitabe chromatic total irregularity index for the Mycielskian graphs of path and cycle. Later, in (Rose and Naduvath, 2020a), they computed the chromatic total irregularity index for path and cycle.

Motivated by the exploration of various coloring-based topological indices, six new chromatic topological indices and ten new induced color-based topological indices are introduced in this article. The induced color-based indices distinguish themselves by providing a unique method to analyze the molecular structures through the color sum of the vertices.

The coloring-based topological indices have not yet been explored especially in the context of their effectiveness in predicting properties through QSPR analysis. To address this research gap, the performance of chromatic topological indices and induced color-based indices are investigated in this article in the context of molecular graph modeling for QSPR analysis. The coloring techniques considered, namely, the proper vertex coloring and sigma coloring have distinct theoretical significance in structural analysis of the molecular structures. The relevance of these coloring techniques and their foundational importance are discussed in detail in the Section 3.1.

Specifically, the induced color-based and chromatic topological indices are computed for 15 potential antivirals of dengue disease. QSPR analysis is performed through these indices and linear regression to explore the physicochemical properties of dengue antivirals. Further, the comparative analysis of the two types of indices is performed to identify the potential indices to predict the properties of drugs.

## 2 Isomorphic molecular graph

The concept of isomorphic molecular graph of a chemical structure is discussed in this section.

### 2.1 Motivation

Wiener (1947) introduced two topological indices, namely, Wiener index and polarity index for alkane molecules. He considered the skeletal structures of alkanes and represented them as molecular graphs to predict their boiling points. In the skeletal structures, the hydrogen atoms bonded to carbon atoms are not explicitly shown. Researchers have extensively computed various topological indices for chemical compounds to predict their properties, often using simplified molecular graph representations. These simplifications typically involve depleting hydrogen atoms and treating double and triple bonds as a single edges. Computing the Wiener index for the simplified molecular graph of a chemical compound does not affect the index value, as the Wiener index is a distance-based topological indices. Later, Ivan Gutman and Oskar E. Polansky (Gutman and Polansky, 2012) introduced the concept of a complete molecular graph, where the molecular graph includes hydrogen atoms but multiple bonds are still represented as single edges. The absence of double or triple bonds in the graph, however, leads to the non-existence of the corresponding chemical structure. Moreover, this simplification particularly affects the degree-based topological indices, as the degree of a vertex varies depending on the multiplicity of its bonds. Consequently, indices calculated using this simplified approach may yield misleading data. These limitations are addressed by W Tamilarasi et al., in (Tamilarasi and Balamurugan, 2024) by introducing an accurate representation of chemical structure as an isomorphic molecular graph. In this representation, double bonds are represented as two parallel edges, triple bonds as three parallel edges and hydrogen atoms are preserved in their adjacency. This approach preserves the unique structural characteristics of the molecule, allowing for accurate comparison and analysis. Therefore, in this article, the isomorphic molecular graph is considered to represent the chemical structure of the potential antivirals of dengue.

Definition 1: (Tamilarasi and Balamurugan, 2024). Let M be a molecular structure of a chemical molecule. The isomorphic molecular graph of M is a graph G(V,E) in which the atoms of M including hydrogen atoms are considered as the vertex set V(G) and the bonds between the atoms in M are considered as the edge set E(G) , where the single bonds are represented as single edge, double bonds as two parallel edges and triple bonds as three parallel edges.

**FIGURE 1:**
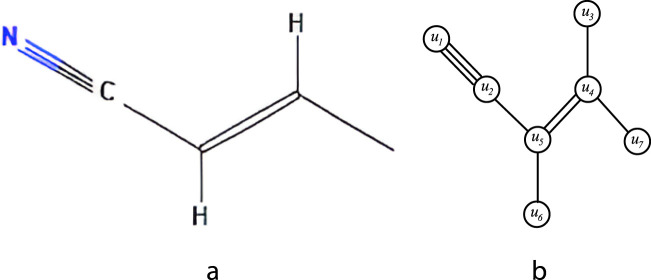
**(a)** Chemical structure of 2-butenenitrile **(b)** Isomorphic molecular graph of 2-butenenitrile.

An example of isomorphic molecular graph is shown in Figure 1.

## 3 Sigma coloring of isomorphic molecular graph and its significance

In this section, the concept of vertex coloring relevant to this article is discussed. Furthermore, the concept of sigma coloring and its significance are presented with appropriate examples.

Definition 2: Let G(V,E) be the isomorphic molecular graph. The vertex coloring c of G is said to be proper vertex coloring if no two adjacent vertices are assigned with the same color.

Definition 3: Let G(V,E) be a graph with vertex set V and edge set E . Let $C=\left\{c_{1},c_{2},\ldots ,c_{\ell }\right\}$ be the set of colors used to color the vertices of G . A $\phi^{-}$ coloring is a vertex coloring in which the colors in C are assigned in an order starting from the first color $c_{1}$ . The color $c_{1}$ is assigned to the maximum possible number of vertices, followed by the color $c_{2}$ is assigned to the maximum possible number of the remaining uncolored vertices. This process is continued sequentially with $c_{3},c_{4},\ldots ,c_{\ell }$ until all vertices in V are colored.

Definition 4: Let G(V,E) be a graph with vertex set V and edge set E . Let $C=\left\{c_{1},c_{2},\ldots ,c_{\ell }\right\}$ be the set of colors used to color the vertices of G . A $\phi^{+}$ coloring is a vertex coloring in which the colors in C are assigned in order starting from the last color $c_{\ell }$ . The color $c_{\ell }$ is assigned to the maximum possible number of vertices, followed by the color $c_{\ell -1}$ is assigned to the maximum possible number of remaining uncolored vertices. This process is continued sequentially with $c_{\ell -2},c_{\ell -3},\ldots ,c_{1}$ until all vertices in V are colored.

Definition 5: Let G(V,E) be a graph with vertex set(atom set) V(G) and edge set (bond set) E(G) . The neighborhood of a vertex(atom) v is defined as the set of vertices(atoms) that are adjacent to v . The cardinality of N(v) denoted as |N(v)| is defined as the number of vertices(atoms) in N(v) .

Definition 6: (Chartrand et al., 2010) Let G(V,E) be a isomorphic molecular graph, where V(G) represents the vertex set(atom set) and E(G) represents the edge set(bond set). Let c:V(G)→N be a vertex(atom) coloring function, where N is a set of natural numbers. The color sum of the atom v∈V(G) , denoted by σ(v) is the sum of colors of the neighbor atoms of v . The coloring c is called sigma coloring if σ(u)≠σ(v) for any two atoms(vertices) u and v that shares a common bond(edge). The minimum number of colors needed for sigma coloring is called as sigma chromatic number of G and it is denoted by σ(G) .

Throughout the article, the values inside the circles represent the proper vertex coloring, while the values outside the circles correspond to the sigma coloring. In sigma coloring, the values outside the brackets is the initial color assigned to the vertex and the values inside the bracket denote the color sum of the vertex.

**FIGURE 2:**
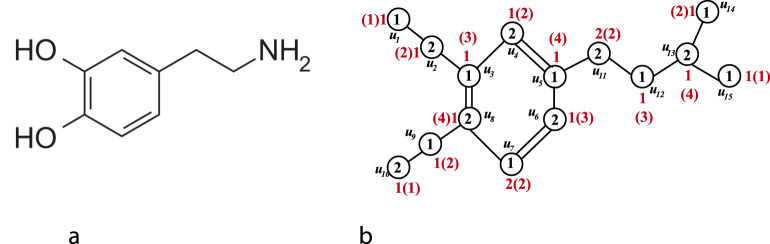
**(a)** Chemical structure of Dopamine **(b)** Vertex coloring and sigma coloring of isomorphic molecular graph of Dopamine.

Example 1: The chemical structure of dopamine is shown in Figure 2a and the vertex coloring and sigma coloring of the isomorphic molecular graph of Dopamine are shown in Figure 2b.

### 3.1 Motivation and significance of sigma coloring

In the sigma coloring of an isomorphic molecular graph G , adjacent atoms can be assigned with the same color. Consequently, atoms of the same type can share the same color to distinguish them from atoms of other types. For example, consider the molecules cyclohexane, cyclohexene, cyclohexadiene and benzene. These molecules differ in their structures based on the number of double bonds present. The chemical structure of these molecules are provided in the Supplementary Material.

Let type 1 carbon atoms be those with a single bond and type 2 carbon atoms be those with a double bond. Cyclohexane contains only type 1 carbon atoms, cyclohexene and cyclohexadiene contain both type 1 and type 2 carbon atoms, while benzene contains only the type 2 carbon atoms. The objective is to distinguish each type of atom through sigma coloring. Initially, the atoms of the isomorphic molecular graph are colored and the color sum of atoms are calculated. If no two adjacent atoms have the same color sum then the type of carbon atom are effectively distinguished based on the assigned colors.

If two adjacent carbon atoms in the isomorphic molecular graphs of cyclohexane, cyclohexene, cyclohexadiene and benzene share a double bond then they are assigned with different colors; otherwise, adjacent carbon atoms are assigned the same color. Hydrogen atoms in the molecule are colored such that the sum of the colors of neighboring atoms of any two adjacent atoms remains distinct.

**FIGURE 3:**
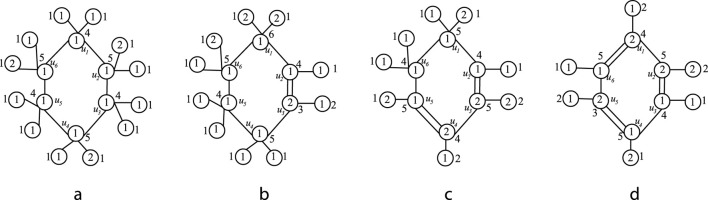
**(a)** Sigma coloring of isomorphic molecular graph of Cyclohexane **(b)** Sigma coloring of isomorphic molecular graph of Cyclohexene **(c)** Sigma coloring of isomorphic molecular graph of Cyclohexadiene **(d)** Sigma coloring of isomorphic molecular graph of benzene.

Let c:V(G)→{1,2} be the vertex coloring of the isomorphic molecular graphs of cyclohexane, cyclohexene, cyclohexadiene and benzene. All atoms are colored in accordance with the aforementioned procedures. The values inside the circle represent the vertex color and the values outside the circle represent the color sum. Observe from Figure 3 that no two adjacent atoms have same color sum. Hence the atoms of the molecules can be differentiated by its types through the sigma coloring. The differentiation of the types are as follows.

Define the cyclic sequence of carbon atom S as $S:c\left(u_{1}\right),c\left(u_{2}\right),c\left(u_{3}\right),c\left(u_{4}\right),c\left(u_{5}\right),c\left(u_{6}\right),c\left(u_{1}\right)$ .

• The cyclic sequence S of cyclohexane is S:1,1,1,1,1,1,1 . From this color sequence, the atoms represented as $u_{1},u_{2},u_{3},u_{4},u_{5},u_{6}$ are the carbon atoms with single bond, since all the atoms are colored with same color.

• The cyclic sequence S of cyclohexene is represented as S:1,1,2,1,1,1,1 . From this color sequence, it is observed that two adjacent pairs of atoms, namely, $u_{2},u_{3}$ and $u_{3},u_{4}$ , are colored with different colors, 1 and 2. This implies the presence of two double bonds. However, carbon atoms can only form up to four covalent bonds. Therefore, there can be only one double bond, and it must be either the pair $\left(u_{2},u_{3}\right)$ or $\left(u_{3},u_{4}\right)$ . In this case, $\left(u_{2},u_{3}\right)$ represents carbon atoms of type 2, while all other atoms are of type 1.

• The cyclic sequence S of cyclohexdiene is S:1,1,2,2,1,1,1 . From this color sequence, it is observed that $u_{2},u_{3}$ and $u_{4},u_{5}$ are colored with different colors and all other pair of atoms are colored with same color. Therefore there exist 2 double bonds.

• The cyclic sequence S of benzene is S:2,2,1,1,2,1,2 . From this color sequence, it can be observed that the atoms in the pairs $\left(u_{2},u_{3}\right),\left(u_{4},u_{5}\right),\left(u_{5},u_{6}\right)$ and $\left(u_{6},u_{1}\right)$ are colored differently. As the same reasoning of cyclohexene, it can be concluded that, $\left(u_{2},u_{3}\right),\left(u_{4},u_{5}\right)$ , and $\left(u_{6},u_{1}\right)$ are of type 2.

From the sigma coloring of cyclohexene and benzene, it is observed that if there is an odd pair of type 2 carbon atoms, then there is a pair of carbon atoms where one is of type 1 and the other is of type 2, and they are colored differently. That is,

• If two adjacent atoms have the same color, then at least one atom among them is of type 1.

• If two atoms have different colors, then both atoms are of type 2.

• In certain cases, such as when an even pair ( k pair) of atoms are colored with different colors, there are either an even number of atoms ( 2k atoms) of type 2 or 2k−2 atoms of type 2.

Unlike other colorings, sigma coloring employs the minimum number of colors. In this approach, any natural numbers can be used to color the atoms(vertices), but the number of colors considered must be minimal. Therefore, the atoms(vertices) in the isomorphic molecular graph can be assigned(colored) with numbers associated with the atoms present in the molecule such as atomic number, mass number oxidation state and so on. For instance, considering the atomic number of all the atoms in a molecule, among them choose the minimum number of colors that satisfies sigma coloring. This shows that sigma coloring allows us to incorporate numerical data associated with the atoms to color the vertices of the graph. Thus, sigma coloring proves to be an effective method for studying molecules.

The following is the general observation of sigma coloring of graphs, which will be used in the proof of the theorem.

Observation 1: (Chartrand et al. 2010) Let G be a molecular graph. Then σ(G)=1 if and only if every two adjacent atoms of G have different degrees.

## 4 Chromatic and induced color-based topological indices

Let G(V,E) be an isomorphic molecular graph, where V represents the atom set and E represents the set of bonds. The ten new induced color-based topological indices and six new chromatic topological indices are introduced in this article and are defined in Table 2.

**TABLE 2:** Induced color-based topological indices and chromatic topological indices with their notations and mathematical formulas.

S.No	Induced color-based topological indices and chromatic topological indices	Notation	Mathematical formula
(i)	First induced color index	$ND_{1}^{\phi_{t}}\left(G\right)$	$\underset{uv\in E\left(G\right)}{\sum }\sqrt{\alpha_{G}\left(u\right)\alpha_{G}\left(v\right)}$
(ii)	Second induced color index	$ND_{2}^{\phi_{t}}\left(G\right)$	$\underset{uv\in E\left(G\right)}{\sum }\frac{1}{\sqrt{\alpha_{G}\left(u\right)+\alpha_{G}\left(v\right)}}$
(iii)	Third induced color index	$ND_{3}^{\phi_{t}}\left(G\right)$	$\underset{uv\in E\left(G\right)}{\sum }\alpha_{G}\left(u\right)\alpha_{G}\left(v\right)\left[\alpha_{G}\left(u\right)+\alpha_{G}\left(v\right)\right]$
(iv)	Fourth induced color index	$ND_{4}^{\phi_{t}}\left(G\right)$	$\underset{uv\in E\left(G\right)}{\sum }\frac{1}{\sqrt{\alpha_{G}\left(u\right)\alpha_{G}\left(v\right)}}$
(v)	Fifth induced color index	$ND_{5}^{\phi_{t}}\left(G\right)$	$\underset{uv\in E\left(G\right)}{\sum }\left[\frac{\alpha_{G}\left(u\right)}{\alpha_{G}\left(v\right)}+\frac{\alpha_{G}\left(v\right)}{\alpha_{G}\left(u\right)}\right]$
(vi)	First induced color Zagreb index	$M_{N}^{\phi_{t}}\left(G\right)$	$\underset{u\in V\left(G\right)}{\sum }{\left[\alpha \left(u\right)\right]}^{2}$
(vii)	Second induced color Zagreb index	$MN_{2}^{\phi_{t}}\left(G\right)$	$\underset{uv\in E\left(G\right)}{\sum }\alpha_{G}\left(u\right)\alpha_{G}\left(v\right)$
(viii)	Forgotten induced color index	$F_{N}^{\phi_{t}}\left(G\right)$	$\underset{u\in V\left(G\right)}{\sum }{\left[\alpha \left(u\right)\right]}^{3}$
(ix)	Modified forgotten induced color index	$MF_{N}^{\phi_{t}}\left(G\right)$	$\underset{uv\in E\left(G\right)}{\sum }\left[{\left(\alpha_{G}\left(u\right)\right)}^{2}+{\left(\alpha_{G}\left(v\right)\right)}^{2}\right]$
(x)	Induced inverse color index	$NI^{\phi_{t}}\left(G\right)$	$\underset{uv\in E\left(G\right)}{\sum }\frac{\alpha_{G}\left(u\right)\alpha_{G}\left(v\right)}{\alpha_{G}\left(u\right)+\alpha_{G}\left(v\right)}$
(xi)	Chromatic Randic index	$R^{\phi_{t}}\left(G\right)$	$\underset{uv\in E\left(G\right)}{\sum }\frac{1}{\sqrt{c\left(u\right)c\left(v\right)}}$
(xii)	Chromatic sum connectivity index	$SC^{\phi_{t}}\left(G\right)$	$\underset{uv\in E\left(G\right)}{\sum }\frac{1}{\sqrt{c\left(u\right)+c\left(v\right)}}$
(xiii)	Chromatic Harmonic index	$H^{\phi_{t}}\left(G\right)$	$\underset{uv\in E\left(G\right)}{\sum }\frac{2}{c\left(u\right)+c\left(v\right)}$
(xiv)	Chromatic forgotten topological index	$F^{\phi_{t}}\left(G\right)$	$\underset{u\in V\left(G\right)}{\sum }\left(c{\left(u\right)}^{3}\right)$
(xv)	Chromatic atom-bond connectivity index	$ABC^{\phi_{t}}\left(G\right)$	$\underset{uv\in E\left(G\right)}{\sum }\sqrt{\frac{c\left(u\right)+c\left(v\right)-2}{c\left(u\right)c\left(v\right)}}$
(xvi)	Chromatic geometric arithmetic index	$GA^{\phi_{t}}\left(G\right)$	$\underset{uv\in E\left(G\right)}{\sum }\frac{2\sqrt{c\left(u\right)c\left(v\right)}}{c\left(u\right)+c\left(v\right)}$

The induced color-based topological indices are computed using the graph coloring variants, where the vertex colors are derived from an initial assignment of vertex or edge colors. Examples of some of such variants include sigma coloring, closed sigma coloring, additive coloring, modular coloring, closed modular coloring, antimagic labeling and lucky labeling. In induced color based topological indices, α(u) represents the induced vertex color. The chromatic topological indices are determined using the proper vertex coloring technique, where c(u) represents the color assigned to the vertex u.

The induced color-based topological indices capture the influence of neighboring vertices or incident edges, whereas chromatic indices utilize graph coloring attributes to represent molecular features.

Let $C=\left\{c_{1},c_{2},\ldots ,c_{l}\right\}$ be the set of colors used to color the vertices of graph that satisfies the condition of the employed coloring. Let each vertex u∈V(G) is assigned a color $c\left(u\right)=c_{j}$ for 1≤j≤l . Let $\phi_{t}:C\to \left\{1,2,\ldots ,l\right\}$ denote the $t^{t}h$ permutation of numbers {1,2,…,l} , for 1≤t≤l! . The value of t ranges from 1 to l! , since ϕ is a permutation function using l colors,. Then, for each such $\phi_{t}$ , the novel induced color-based topological indices and chromatic topological indices are defined in Table 2.

Let $C=\left\{c_{1},c_{2},\ldots ,c_{l}\right\}$ be a proper vertex coloring of a graph G , where each vertex u∈V(G) is assigned a color $c\left(u\right)=c_{j}$ for 1≤j≤l . Let $\phi_{t}:C\to \left\{1,2,\ldots ,l\right\}$ denote the $t^{t}h$ permutation of numbers {1,2,…,l} , for 1≤t≤l! . The value of t ranges from 1 to l! , since ϕ is a permutation function using l colors. Then, for each such $\phi_{t}$ , the chromatic topological indices introduced by Kok et al. (2016) and Rose and Naduvath (2020a) are defined as follows:

1. The first chromatic Zagreb index of G is defined as, $M_{1}^{\phi_{t}}$ = $\sum_{j=1}^{l}\theta \left(c_{j}\right)j^{2}$ , where $\theta \left(c_{j}\right)$ is the number of vertices colored with the color $c_{j}$ in G .
2. The second chromatic Zagreb index of G is defined as, $M_{2}^{\phi_{t}}$ = $\sum_{uv\in E\left(G\right)}c\left(u\right)c\left(v\right)$

3. The chromatic irregularity index of G is defined as, $M_{3}^{\phi_{t}}$ = $\sum_{uv\in E\left(G\right)}|c\left(u\right)-c\left(v\right)|$

4. The chromatic total irregularity index of G is defined as, $M_{4}^{\phi_{t}}$ = $\sum_{uv\in E\left(G\right)}\frac{1}{2}|c\left(u\right)-c\left(v\right)|$

Let $\phi^{-}$ denote the permutation $\phi_{t}$ that yields the minimum value of the topological index among all l! possible permutations and let $\phi^{+}$ refers to the permutation $\phi_{t}$ that gives the maximum value. Let InV be the topological index value correspond to the given permutation. Then, the relationship between the topological index value is given by

$$InV^{\phi^{-}}\leq InV^{\phi_{t}}\leq InV^{\phi^{+}}$$

These topological indices form the basis for QSPR analysis to improve the predictive accuracy of analysis in determining the physicochemical properties of antiviral drugs of dengue. The induced color-based topological indices are computed using a graph coloring variant known as sigma coloring, while the chromatic topological indices are calculated using the proper vertex coloring.

## 5 Methodology

A systematic approach is employed to analyze the properties of potential antiviral drugs for dengue disease through the Quantitative Structure-Property Relationship (QSPR) graph modelling by considering the topological descriptors of the isomorphic molecular graphs of the drugs. The methodology consists of the following key steps:

1. Data Collection and Isomorphic Molecular Graph Construction
  Potential antiviral drugs for dengue were selected based on their efficacy. The molecular structures of these drugs were obtained from publicly available database PubChem whose URL is pubchem.ncbi.nlm.nih.gov. Each chemical structure was then modelled as an isomorphic molecular graph.
2. Computation of Topological Indices
  The chromatic and induced color-based topological indices of the molecular graphs were computed through two distinct coloring approaches namely, proper vertex coloring and sigma coloring respectively.
3. QSPR Analysis
  The QSPR analysis was conducted to explore the relationship between the computed topological indices of molecular graphs and physicochemical properties of antiviral drugs used for dengue treatment. Linear regression analysis was performed using the Statistical Package for the Social Sciences (SPSS) software, applying statistical methods to evaluate the strength and significance of these correlations.
4. Statistical Analysis and Descriptor Evaluation
  The predictive capability of the computed indices was assessed using the following statistical parameters:

• Correlation coefficient $\left(R^{2}\right)$ : Measures the strength of association between variables.

• Significance tests ( p -values): Determine the reliability of the observed relationships.

• Standard error values: Evaluate the accuracy and precision of the model.

• Y-randomization test: Ensures that the QSPR analysis results are not obtained by chance.

The most effective indices were identified based on their predictive power and statistical significance, offering a robust framework for understanding the molecular properties of antiviral drugs used in dengue treatment.

## 6 Computation of chromatic and induced color-based topological indices of potential antivirals of dengue

In this section, the $\phi^{-}$ and $\phi^{+}$ chromatic and induced color-based topological indices of potential antivirals of dengue disease are computed through proper vertex coloring and sigma coloring respectively. The two coloring scheme $\phi^{-}$ and $\phi^{+}$ are employed to investigate how the order of color assignment influence the predictive power of the topological indices. The chemical structure of the considered potential antivirals of the dengue disease are obtained from the National Center for Biotechnology Information (NCBI) whose URL is pubchem.ncbi.nlm.nih.gov and they are provided in the Supplementary Material.

### 6.1 Computation of chromatic topological indices

Let G(V,E) be an isomorphic molecular graph with vertex set V(G) and edge set E(G) and let $c:V\left(G\right)\to \left\{c_{1},c_{2},\ldots ,c_{l}\right\}$ be the vertex coloring of G . The following steps are followed to color the vertices of the isomorphic molecular graphs of potential antivirals of dengue such that the vertex coloring is a proper vertex coloring.

1. Partition the vertices into independent sets.
2. Compute $\phi^{-}$ proper vertex coloring of G .
    The color $c_{1}$ is assigned to the vertices in the independent set with the highest cardinality, followed by the color $c_{2}$ , which is assigned to the vertices in the independent set with the second-highest cardinality. This process is repeated until all vertices of the graph are colored, ensuring that the coloring satisfies the condition of proper vertex coloring.
3. Compute the $\phi^{+}$ proper vertex coloring of G .
    The color $c_{l}$ is assigned to the vertices in the independent set with the highest cardinality, followed by the color $c_{l-1}$ , which is assigned to the vertices in the independent set with the second-highest cardinality. This process is repeated until all vertices of the graph are colored, ensuring that the coloring satisfies the condition of proper vertex coloring.

Theorem 1: Let G be the isomorphic molecular graph of Lycorine. The chromatic topological indices of G through $\phi^{-}$ and $\phi^{+}$ proper vertex coloring are as follows:

$M_{1}^{\phi^{-}}\left(G\right)=74,\;M_{2}^{\phi^{-}}\left(G\right)=69,\;M_{3}^{\phi^{-}}\left(G\right)=32,\;M_{4}^{\phi^{-}}\left(G\right)=16$ , $R^{\phi^{-}}\left(G\right)=19.519,\;SC^{\phi^{-}}\left(G\right)=16.251,\;H^{\phi^{-}}\left(G\right)=18.3,\;F^{\phi^{-}}\left(G\right)=154,$ $ABC^{\phi^{-}}\left(G\right)=20.834,GA^{\phi^{-}}\left(G\right)=27.168.\;\;M_{1}^{\phi^{+}}\left(G\right)=154,M_{2}^{\phi^{+}}\left(G\right)=157,$ $M_{3}^{\phi^{+}}\left(G\right)=32,\;M_{4}^{\phi^{+}}\left(G\right)=16,\;R^{\phi^{+}}\left(G\right)=12.944,SC^{\phi^{+}}\left(G\right)=13.388,$ $H^{\phi^{+}}\left(G\right)=12.433,F^{\phi^{+}}\left(G\right)=414,ABC^{\phi^{+}}\left(G\right)=20.834,\;GA^{\phi^{+}}\left(G\right)=27.999$ .

**FIGURE 4:**
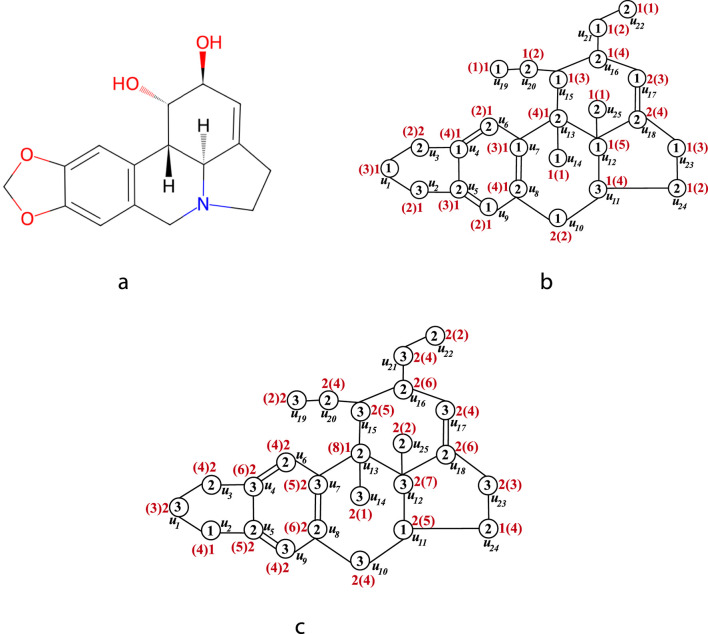
**(a)** The chemical structure of lycorine **(b)** The $\phi^{-}$ vertex coloring and $\phi^{-}$ sigma coloring of the isomorphic molecular graph of lycorine. **(c)** The $\phi^{+}$ vertex coloring and $\phi^{+}$ sigma coloring of the isomorphic molecular graph of lycorine.

Proof. Let G be the isomorphic molecular graph of Lycorine representing the 25 atoms and 29 bonds as vertices and edges respectively. Let $V\left(G\right)=\left\{u_{1},u_{2},\ldots ,u_{25}\right\}$ be the atom(vertex) set of G . The chemical structure of lycorine is shown in Figure 4a. The independent sets of the graph G are as follows:

$$\begin{array}{c} A_{1}=\left\{u_{1},u_{4},u_{7},u_{9},u_{10},u_{12},u_{14},u_{15},u_{17},u_{19},u_{21},u_{23}\right\},\\ A_{2}=\left\{u_{3},u_{5},u_{6},u_{8},u_{13},u_{16},u_{18},u_{20},u_{22},u_{24},u_{25}\right\},\\ A_{3}=\left\{u_{2},u_{11}\right\}. \end{array}$$

The graph G can be colored with three colors because the vertices of G can be partitioned into three independent sets. Let c:V(G)→{1,2,3} be the vertex coloring of G . From the sets A_1_, A_2_ and A_3_, it is observed that $|A_{1}|=12,\;|A_{2}|=11$ and $|A_{3}|=2$ . Thus, $\left|A_{1}|>|A_{2}|>|A_{3}\right|$ . Now we have to prove that c is a proper vertex coloring of G .

#### 6.1.1 Case 1. $\phi^{-}$ coloring

In the case of $\phi^{-}$ coloring, the vertices in $A_{1},A_{2}$ and $A_{3}$ are colored with the colors 1, 2 and 3 respectively. This coloring yields the proper vertex coloring. The $\phi^{-}$ proper vertex coloring of G is shown in Figure 4b. Using these proper vertex color, the chromatic topological indices of G are computed.

The number of vertices colored 1, 2 and 3 are 12, 11 and 2 respectively. Similarly, the number of end vertices of an edge with the color pair (1,2), (1,3) and (2,3) are 24, 3 and 2 respectively. Using the mathematical expressions presented in the Table 2 and in Section 4, the following chromatic topological indices are computed.

$$\begin{array}{ll} M_{1}^{\phi^{-}}\left(G\right) & =\overset{l}{\underset{j=1}{\sum }}\theta \left(c_{j}\right)j^{2}=12\left(1^{2}\right)+11\left(2^{2}\right)+2\left(3^{2}\right)=74.\\ M_{2}^{\phi^{-}}\left(G\right) & =\underset{uv\in E\left(G\right)}{\sum }c\left(u\right)c\left(v\right)=24\left(2\right)+3\left(3\right)+2\left(6\right)=69.\\ M_{3}^{\phi^{-}}\left(G\right) & =\underset{uv\in E\left(G\right)}{\sum }|c\left(u\right)-c\left(v\right)|=24\left(1\right)+3\left(2\right)+2\left(1\right)=32.\\ M_{4}^{\phi^{-}}\left(G\right) & =\underset{uv\in E\left(G\right)}{\sum }\frac{1}{2}|c\left(u\right)-c\left(v\right)|=12+3+1=16\\ R^{\phi^{-}}\left(G\right) & =\underset{uv\in E\left(G\right)}{\sum }\frac{1}{\sqrt{c\left(u\right)c\left(v\right)}}=\frac{24}{\sqrt{2}}+\frac{3}{\sqrt{3}}+\frac{2}{\sqrt{6}}=19.519\\ SC^{\phi^{-}}\left(G\right) & =\underset{uv\in E\left(G\right)}{\sum }\frac{1}{\sqrt{c\left(u\right)+c\left(v\right)}}=\frac{24}{\sqrt{3}}+\frac{3}{\sqrt{4}}+\frac{2}{\sqrt{5}}=16.251\\ H^{\phi^{-}}\left(G\right) & =\underset{uv\in E\left(G\right)}{\sum }\frac{2}{c\left(u\right)+c\left(v\right)}=\frac{2\left(24\right)}{3}+\frac{2\left(3\right)}{4}+\frac{2\left(2\right)}{5}=18.3\\ F^{\phi^{-}}\left(G\right) & =\underset{u\in V\left(G\right)}{\sum }\left(c{\left(u\right)}^{3}\right)=12\left(1^{3}\right)+11\left(2^{3}\right)+2\left(3^{3}\right)=154\\ ABC^{\phi^{-}}\left(G\right) & =\underset{uv\in E\left(G\right)}{\sum }\sqrt{\frac{c\left(u\right)+c\left(v\right)-2}{c\left(u\right)c\left(v\right)}}=24\sqrt{\frac{1}{2}}+3\sqrt{\frac{2}{3}}+2\sqrt{\frac{3}{6}}=20.834\\ GA^{\phi^{-}}\left(G\right) & =\underset{uv\in E\left(G\right)}{\sum }\frac{2\sqrt{c\left(u\right)c\left(v\right)}}{c\left(u\right)+c\left(v\right)}=2\left(\frac{24\sqrt{2}}{3}\right)+2\left(\frac{3\sqrt{3}}{4}\right)+2\left(\frac{2\sqrt{6}}{5}\right)=27.168 \end{array}$$

#### 6.1.2 Case 2. $\phi^{+}$ coloring

In the case of $\phi^{+}$ coloring, the vertices in $A_{1},A_{2}$ and $A_{3}$ are colored with the colors 3, 2 and 1 respectively. This coloring yields the proper vertex coloring. The $\phi^{+}$ proper vertex coloring is shown in Figure 4c. Using these vertex colors, the chromatic topological indices of G are computed.

The number of vertices colored 1, 2 and 3 are 2, 11 and 12 respectively. Similarly, the number of end vertices of an edge with the color pairs (1,2), (1,3) and (2,3) are 2, 3 and 24 respectively. Using the mathematical expressions presented in the Table 2 and in Section 4, the following chromatic topological indices are computed.

$$\begin{array}{ll} M_{1}^{\phi^{+}}\left(G\right) & =\overset{l}{\underset{j=1}{\sum }}\theta \left(c_{j}\right)j^{2}=12\left(9\right)+11\left(4\right)+2=154\\ M_{2}^{\phi^{+}}\left(G\right) & =\underset{uv\in E\left(G\right)}{\sum }c\left(u\right)c\left(v\right)=24\left(6\right)+3\left(3\right)+2\left(2\right)=157\\ M_{3}^{\phi^{+}}\left(G\right) & =\underset{uv\in E\left(G\right)}{\sum }|c\left(u\right)-c\left(v\right)|=24\left(1\right)+3\left(2\right)+2\left(1\right)=32\\ M_{4}^{\phi^{+}}\left(G\right) & =\underset{uv\in E\left(G\right)}{\sum }\frac{1}{2}|c\left(u\right)-c\left(v\right)|=12+3+1=16\\ R^{\phi^{+}}\left(G\right) & =\underset{uv\in E\left(G\right)}{\sum }\frac{1}{\sqrt{c\left(u\right)c\left(v\right)}}=\frac{24}{\sqrt{6}}+\frac{3}{\sqrt{3}}+\frac{2}{\sqrt{2}}=12.944\\ SC^{\phi^{+}}\left(G\right) & =\underset{uv\in E\left(G\right)}{\sum }\frac{1}{\sqrt{c\left(u\right)+c\left(v\right)}}=\frac{24}{\sqrt{5}}+\frac{3}{\sqrt{4}}+\frac{2}{\sqrt{3}}=13.388\\ H^{\phi^{+}}\left(G\right) & =\underset{uv\in E\left(G\right)}{\sum }\frac{2}{c\left(u\right)+c\left(v\right)}=\frac{48}{5}+\frac{6}{4}+\frac{4}{3}=12.433\\ F^{\phi^{+}}\left(G\right) & =\underset{u\in V\left(G\right)}{\sum }\left(c{\left(u\right)}^{3}\right)=12\left(3^{3}\right)+11\left(2^{3}\right)+2\left(1^{3}\right)=414\\ ABC^{\phi^{+}}\left(G\right) & =\underset{uv\in E\left(G\right)}{\sum }\sqrt{\frac{c\left(u\right)+c\left(v\right)-2}{c\left(u\right)c\left(v\right)}}=24\sqrt{\frac{3}{6}}+3\sqrt{\frac{2}{3}}+2\sqrt{\frac{1}{2}}=20.834\\ GA^{\phi^{+}}\left(G\right) & =\underset{uv\in E\left(G\right)}{\sum }\frac{2\sqrt{c\left(u\right)c\left(v\right)}}{c\left(u\right)+c\left(v\right)}=2\left(\frac{24\sqrt{6}}{5}\right)+2\left(\frac{3\sqrt{3}}{4}\right)+2\left(\frac{2\sqrt{2}}{3}\right)=27.999 \end{array}$$

In a similar manner, the chromatic topological indices for the isomorphic molecular graphs of UV-4B (N-9-methoxynonyl-1-deoxynojirimycin), ST-148, 4-HPR, Silymarin, Baicalein, Quercetin, Naringenin, Nelfinavir, Ivermectin, Mosnodenvir (JNJ-1802), NITD-688, Metoclopramide, JNJ-A07 and Betulinic acid are computed. The $\phi^{-}$ and $\phi^{+}$ proper vertex coloring of the isomorphic molecular graphs of the considered potential antivirals of dengue disease are provided in the Supplementary Material and their computed chromatic topological indices are tabulated in Table 3.

**TABLE 3:** The computed chromatic topological indices through $\phi^{-}$ and $\phi^{+}$ proper vertex coloring of 15 potential antivirals of dengue disease.

Chromatic topological indices values obtained through $\phi^{-}$ proper vertex coloring										
Drugs	$M_{1}^{\phi^{-}}\left(G\right)$	$M_{2}^{\phi^{-}}\left(G\right)$	$M_{3}^{\phi^{-}}\left(G\right)$	$M_{4}^{\phi^{-}}\left(G\right)$	$R^{\phi^{-}}\left(G\right)$	$SC^{\phi^{-}}\left(G\right)$	$H^{\phi^{-}}\left(G\right)$	$F^{\phi^{-}}\left(G\right)$	$ABC^{\phi^{-}}\left(G\right)$	$GA^{\phi^{-}}\left(G\right)$
Lycorine	74	69	32	16	19.519	16.251	18.3	154	20.834	27.168
UV-4B	65	52	26	13	18.385	15.011	17.333	117	18.385	24.513
ST-148	94	88	39	19.5	25.007	20.817	23.533	94	49.826	34.841
4-HPR	88	76	38	19	26.87	21.939	25.333	156	26.870	35.827
Silymarin	97	88	44	22	31.113	25.403	29.333	173	31.113	41.484
Baicalein	56	50	25	12.5	17.678	14.434	16.667	100	17.678	23.570
Quercetin	40	58	29	14.5	20.506	16.743	19.333	118	20.506	27.341
Naringenin	56	50	25	12.5	17.678	14.434	16.667	100	17.678	23.570
Nelfinavir	112	98	49	24.5	34.648	28.29	32.667	200	34.648	46.198
Ivermectin	185	170	83	41.5	54.745	45.041	51.467	349	56.299	74.249
Mosnodenvir	97	97	45	22.5	30.385	25.066	28.633	193	31.222	41.481
NITD-688	90	86	42	21	27.726	22.809	26.067	182	28.503	37.596
Metoclopramide	53	46	23	11.5	16.263	13.279	15.333	93	16.263	21.685
JNJ-A07	101	96	47	23.5	31.261	25.696	29.4	201	32.039	42.310
Betulinic acid	95	95	44	22	29.678	24.488	27.967	191	2.938	2.938

Chromatic topological indices values obtained through $\phi^{+}$ proper vertex coloring										
Drugs	$M_{1}^{\phi^{+}}\left(G\right)$	$M_{2}^{\phi^{+}}\left(G\right)$	$M_{3}^{\phi^{+}}\left(G\right)$	$M_{4}^{\phi^{+}}\left(G\right)$	$R^{\phi^{+}}\left(G\right)$	$SC^{\phi^{+}}\left(G\right)$	$H^{\phi^{+}}\left(G\right)$	$F^{\phi^{+}}\left(G\right)$	$ABC^{\phi^{+}}\left(G\right)$	$GA^{\phi^{+}}\left(G\right)$
Lycorine	154	157	32	16	12.94	13.39	12.43	414	20.83	28
UV-4B	65	52	26	13	18.39	15.01	17.33	117	18.39	24.51
ST-148	206	204	39	19.5	16.34	17.04	15.8	554	26.38	35.91
4-HPR	97	76	38	19	26.87	65.82	25.33	177	26.87	35.83
Silymarin	103	88	44	22	31.11	25.4	29.33	187	31.11	41.48
Baicalein	59	50	25	12.5	17.68	14.43	16.67	107	17.68	23.57
Quercetin	69	58	29	14.5	20.51	16.74	19.33	45	20.51	27.34
Naringenin	59	50	25	12.5	17.68	14.43	16.67	107	17.68	23.57
Nelfinavir	118	98	49	24.5	34.65	28.29	32.67	214	69.3	46.2
Ivermectin	481	454	83	41.5	35.53	35.8	32.53	1311	56.3	56.5
Mosnodenvir	271	253	45	22.5	18.73	19.99	18.23	739	31.22	42.92
NITD-688	240	230	42	21	16.97	18.12	16.47	650	28.5	38.93
Metoclopramide	62	46	23	11.5	16.26	7.67	15.33	114	16.26	21.69

### 6.2 Computation of induced color-based topological indices

Let $c:V\left(G\right)\to \left\{c_{1},c_{2},\ldots ,c_{l}\right\}$ be the vertex coloring of G . The following steps are followed to color the vertices of isomorphic molecular graph of the considered antivirals of dengue, such that the vertex coloring c is a sigma coloring.

1. If two adjacent vertices, say u and v , have different degrees, then the vertices in N(u) and N(v) can be colored with the same color such that σ(u)≠σ(v) .
2. If two adjacent vertices, say u and v , have equal degree, then atleast one vertex in N(u) or N(v) must be assigned with the different color such that σ(u)≠σ(v) .

Theorem 2: Let G be the isomorphic molecular graph of Lycorine. The induced color based topological indices of the graph G through $\phi^{-}$ and $\phi^{+}$ sigma coloring are as follows:

$ND_{1}^{\phi^{-}}\left(G\right)=85.139,\;ND_{2}^{\phi^{-}}\left(G\right)=12.022,\;ND_{3}^{\phi^{-}}\left(G\right)=1820,$ $ND_{4}^{\phi^{-}}\left(G\right)=10.674,ND_{5}^{\phi^{-}}\left(G\right)=69.933,\;M_{N}^{\phi^{-}}\left(G\right)=211,$ $MN_{2}^{\phi^{-}}\left(G\right)=267,\;F_{N}^{\phi^{-}}\left(G\right)=739,MF_{N}^{\phi^{-}}\left(G\right)=607\;$ and $NI^{\phi^{-}}\left(G\right)=41.081$

$ND_{1}^{\phi^{+}}\left(G\right)=136.948,\;ND_{2}^{\phi^{+}}\left(G\right)=9.447,\;ND_{3}^{\phi^{+}}\left(G\right)=6104,$ $ND_{4}^{\phi^{+}}\left(G\right)=6.367,\;ND_{5}^{\phi^{+}}\left(G\right)=69.833,\;M_{N}^{\phi^{+}}\left(G\right)=532,$ $MN_{2}^{\phi^{+}}\left(G\right)=683,\;F_{N}^{\phi^{+}}\left(G\right)=2874,MF_{N}^{\phi^{+}}\left(G\right)=1521\;and\;NI^{\phi^{+}}\left(G\right)=66.604$ .

Proof. Let G be the isomorphic molecular graph of Lycorine where 25 atoms and 29 bonds are represented as vertices and edges respectively. Let $V\left(G\right)=\left\{u_{1},u_{2},\ldots ,u_{25}\right\}$ be the vertex set of G . The chemical structure of lycorine is shown in Figure 4a. By Observation 1, we have the inequality.

$$\sigma \left(G\right)\geq \;2.$$

Let c:V(G)→{1,2} be the vertex coloring of G . Now we have to prove that c is a sigma coloring of G .

From the isomorphic molecular graph of G , it is observed that $d\left(u_{1}\right)=d\left(u_{3}\right),\;d\left(u_{1}\right)=d\left(u_{2}\right),d\left(u_{4}\right)=d\left(u_{5}\right),d\left(u_{7}\right)=d\left(u_{8}\right),$ $d\left(u_{12}\right)=d\left(u_{13}\right),d\left(u_{15}\right)=d\left(u_{16}\right)\;and\;d\left(u_{23}\right)=d\left(u_{24}\right)$ . By following the steps 1 and 2, the vertices with the same degree and different degrees are assigned colors as shown in Table 4.

**TABLE 4:** The vertex coloring and color sum of the vertices for $\phi^{-}$ and $\phi^{+}$ sigma coloring of G .

The vertex coloring and color sum of the adjacent vertices having same degree					
Vertex u	N(u)	Vertex color of N(u)		Color sum of the vertex u (α(u))	
		$\phi^{-}$	$\phi^{+}$	$\phi^{-}$	$\phi^{+}$
$u_{1}$	$\left(u_{2},u_{3}\right)$	(1,2)	(1,2)	3	3
$u_{23}$	$\left(u_{24},u_{18}\right)$				
$u_{2}$	$\left(u_{1},u_{5}\right)$	(1,1)	(2,2)	2	4
$u_{3}$	$\left(u_{1},u_{4}\right)$				
$u_{24}$	$\left(u_{11},u_{23}\right)$				
$u_{4}$	$\left(u_{5},u_{6},u_{3}\right)$	(1,1,2)	(2,2,2)	4	6
$u_{8}$	$\left(u_{7},u_{9},u_{10}\right)$				
$u_{16}$	$\left(u_{15},u_{21},u_{17}\right)$				
$u_{5}$	$\left(u_{2},u_{4},u_{9}\right)$	(1,1,1)	(1,2,2)	3	5
$u_{7}$	$\left(u_{13},u_{8},u_{6}\right)$				
$u_{15}$	$\left(u_{13},u_{16},u_{20}\right)$				
$u_{12}$	$\left(u_{11},u_{13},u_{18},u_{25}\right)$	(1,1,2,1)	(2,1,2,2)	5	7
$u_{13}$	$\left(u_{7},u_{12},u_{14},u_{15}\right)$	(1,1,1,1)	(2,2,2,2)	4	8

The vertex coloring and color sum of the remaining vertices					
Vertex u	N(u)	Vertex color of N(u)		Color sum of the vertex u (α(u))	
		$\phi^{-}$	$\phi^{+}$	$\phi^{-}$	$\phi^{+}$
$u_{6}$	$\left(u_{4},u_{7}\right)$	(1,1)	(2,2)	2	4
$u_{9}$	$\left(u_{5},u_{8}\right)$				
$u_{10}$	$\left(u_{8},u_{11}\right)$				
$u_{20}$	$\left(u_{15},u_{19}\right)$				
$u_{21}$	$\left(u_{16},u_{22}\right)$				
$u_{19}$	$\left(u_{20}\right)$	(1)	(2)	1	2
$u_{22}$	$\left(u_{21}\right)$				
$u_{25}$	$\left(u_{12}\right)$				
$u_{11}$	$\left(u_{10},u_{12},u_{24}\right)$	(2,1,1)	(2,2,1)	4	5
$u_{18}$	$\left(u_{12},u_{17},u_{23}\right)$	(1,2,1)	(2,2,2)	4	6
$u_{17}$	$\left(u_{16},u_{18}\right)$	(1,2)	(2,2)	3	4
$u_{14}$	$\left(u_{13}\right)$	(1)	(1)	1	1

By comparing the α(u) values in Table 4 for any two adjacent vertices in G , it is observed that adjacent vertices with the same degree and distinct degree have distinct color sums. Thus c is both $\phi^{-}$ sigma coloring and $\phi^{+}$ sigma coloring. The $\phi^{-}$ and $\phi^{+}$ sigma coloring of G are shown in Figures 4b,c respectively.

Using the α(u) value from Table 4, the induced color based topological indices are calculated for both $\phi^{-}$ coloring and $\phi^{+}$ coloring. Table 5 present the values necessary for the efficient computation of induced color-based topological indices in $\phi^{-}$ sigma coloring.

**TABLE 5:** The vertex and edge distribution based on color sums of vertices in $\phi^{-}$ and $\phi^{+}$ sigma coloring of Lycorine.

(a) Vertex distribution for $\phi^{-}$ sigma coloring of Lycorine.					
$\phi^{-}$ sigma coloring					
Color Sum α(u)	1	2	3	4	5
Number of Vertices	4	8	6	6	1

(b) Vertex distribution for $\phi^{+}$ sigma coloring of Lycorine.								
$\phi^{+}$ sigma coloring								
Color Sum α(u)	1	2	3	4	5	6	7	8
Number of Vertices	1	3	2	9	4	4	1	1

(c) Edge distribution for $\phi^{-}$ sigma coloring of Lycorine.							
$\phi^{-}$ sigma coloring							
Color sums of end vertices (α(u),α(v))	(1,2)	(1,4)	(1,5)	(2,3)	(2,4)	(3,4)	(4,5)
Number of Edges	2	1	1	7	7	8	3

(d) Edge distribution for $\phi^{+}$ sigma coloring of Lycorine.												
$\phi^{+}$ sigma coloring												
Color sums of end vertices (α(u),α(v))	(3,4)	(4,6)	(4,5)	(6,5)	(5,8)	(7,8)	(8,1)	(7,5)	(6,7)	(7,2)	(6,3)	(4,2)
Number of Edges	3	7	6	3	2	1	1	1	1	1	1	2

The following are the induced color-based indices of G obtained through $\phi^{-}$ sigma coloring.

$$\begin{array}{ll} ND_{1}^{\phi^{-}}\left(G\right) & =\underset{uv\in E\left(G\right)}{\sum }\sqrt{\alpha \left(u\right)\alpha \left(v\right)}\\ & =2\sqrt{2}+1\sqrt{4}+\sqrt{5}+7\sqrt{6}+7\sqrt{8}+8\sqrt{12}+3\sqrt{20}\\ & =85.139 \end{array}$$

$$\begin{array}{ll} ND_{2}^{\phi^{-}}\left(G\right) & =\underset{uv\in E\left(G\right)}{\sum }\frac{1}{\sqrt{\alpha \left(u\right)+\alpha \left(v\right)}}\\ & =\frac{2}{\sqrt{3}}+\frac{1}{\sqrt{5}}+\frac{1}{\sqrt{6}}+\frac{7}{\sqrt{5}}+\frac{7}{\sqrt{6}}+\frac{8}{\sqrt{7}}+\frac{3}{\sqrt{9}}\\ & =12.022\\ ND_{3}^{\phi^{-}}\left(G\right) & =\underset{uv\in E\left(G\right)}{\sum }\alpha \left(u\right)\alpha \left(v\right)\left[\alpha \left(u\right)+\alpha \left(v\right)\right]\\ & =\left(2\times 2\times 3\right)+\left(1\times 4\times 5\right)+\left(1\times 5\times 6\right)\\ & +\left(7\times 6\times 5\right)+\left(7\times 8\times 6\right)+\left(8\times 12\times 7\right)\\ & +\left(3\times 20\times 9\right).\\ & =1820\\ ND_{4}^{\phi^{-}}\left(G\right) & =\underset{uv\in E\left(G\right)}{\sum }\frac{1}{\sqrt{\alpha \left(u\right)\alpha \left(v\right)}}\\ & =\frac{2}{\sqrt{2}}+\frac{1}{\sqrt{4}}+\frac{1}{\sqrt{5}}+\frac{7}{\sqrt{6}}+\frac{7}{\sqrt{8}}+\frac{8}{\sqrt{12}}+\frac{3}{\sqrt{20}}\\ & =10.674 \end{array}$$

$$\begin{array}{ll} ND_{5}^{\phi^{-}}\left(G\right) & =\underset{uv\in E\left(G\right)}{\sum }\left[\frac{\alpha \left(u\right)}{\alpha \left(v\right)}+\frac{\alpha \left(v\right)}{\alpha \left(u\right)}\right]\\ & =2\left[\frac{1}{2}+\frac{2}{1}\right]+1\left[\frac{1}{4}+\frac{4}{1}\right]+\left[\frac{1}{5}+\frac{5}{1}\right]+7\left[\frac{2}{3}+\frac{3}{2}\right]+8\left[\frac{3}{4}+\frac{4}{3}\right]\\ & =+3\left[\frac{4}{5}+\frac{5}{4}\right]+7\left[\frac{2}{4}+\frac{4}{2}\right]\\ & =69.933\\ M_{N}^{\phi^{-}}\left(G\right) & =\underset{u\in V\left(G\right)}{\sum }{\left(\alpha \left(u\right)\right)}^{2}\\ & =4\left(1^{2}\right)+8\left(2^{2}\right)+6\left(3^{2}\right)+6\left(4^{2}\right)+1\left(5^{2}\right)\\ & =211\\ MN_{2}^{\phi^{-}}\left(G\right) & =\underset{uv\in E\left(G\right)}{\sum }\alpha \left(u\right)\alpha \left(v\right)\\ & =2\left(2\right)+1\left(4\right)+1\left(5\right)+7\left(6\right)+7\left(8\right)+8\left(12\right)+3\left(20\right)\\ & =267\\ F_{N}^{\phi^{-}}\left(G\right) & =\underset{u\in V\left(G\right)}{\sum }{\left(\alpha \left(u\right)\right)}^{3}\\ & =4\left(1^{3}\right)+8\left(2^{3}\right)+6\left(3^{3}\right)+6\left(4^{3}\right)+1\left(5^{3}\right)\\ & =739 \end{array}$$

$$\begin{array}{ll} MF_{N}^{\phi^{-}}\left(G\right) & =\underset{uv\in E\left(G\right)}{\sum }\left(\alpha {\left(u\right)}^{2}+\alpha {\left(v\right)}^{2}\right)\\ & =2\left(1^{2}+2^{2}\right)+1\left(1^{2}+4^{2}\right)+1\left(1^{2}+5^{2}\right)+7\left(2^{2}+3^{2}\right)\\ & +7\left(2^{2}+4^{2}\right)+8\left(3^{2}+4^{2}\right)+3\left(4^{2}+5^{2}\right)\\ & =607\\ NI^{\phi^{-}}\left(G\right) & =\underset{uv\in E\left(G\right)}{\sum }\frac{\alpha \left(u\right)\alpha \left(v\right)}{\alpha \left(u\right)+\alpha \left(v\right)}\\ & =2\left(\frac{1\times 2}{1+2}\right)+\left(\frac{1\times 4}{1+4}\right)+\left(\frac{1\times 5}{1+5}\right)+7\left(\frac{2\times 3}{2+3}\right)\\ & +7\left(\frac{2\times 4}{2+4}\right)+8\left(\frac{3\times 4}{3+4}\right)+3\left(\frac{4\times 5}{4+5}\right)\\ & =41.081 \end{array}$$

The Table 5 presents the values necessary for the efficient computation of induced color-based topological indices in $\phi^{+}$ sigma coloring.

The following are the induced color-based indices of G obtained through $\phi^{+}$ sigma coloring of G .

$$\begin{array}{ll} ND_{1}^{\phi^{+}}\left(G\right) & =\underset{uv\in E\left(G\right)}{\sum }\sqrt{\sigma \left(u\right)\sigma \left(v\right)}=136.948\\ ND_{2}^{\phi^{+}}\left(G\right) & =\underset{uv\in E\left(G\right)}{\sum }\frac{1}{\sqrt{\sigma \left(u\right)+\sigma \left(v\right)}}=9.447\\ ND_{3}^{\phi^{+}}\left(G\right) & =\underset{uv\in E\left(G\right)}{\sum }\sigma \left(u\right)\sigma \left(v\right)\left[\sigma \left(u\right)+\sigma \left(v\right)\right]=6104\\ ND_{4}^{\phi^{+}}\left(G\right) & =\underset{uv\in E\left(G\right)}{\sum }\frac{1}{\sqrt{\sigma \left(u\right)\sigma \left(v\right)}}=6.367\\ ND_{5}^{\phi^{+}}\left(G\right) & =\underset{uv\in E\left(G\right)}{\sum }\left[\frac{\sigma \left(u\right)}{\sigma \left(v\right)}+\frac{\sigma \left(v\right)}{\sigma \left(u\right)}\right]=69.833\\ M_{N}^{\phi^{+}}\left(G\right) & =\underset{u\in V\left(G\right)}{\sum }{\left(\sigma \left(u\right)\right)}^{2}=532\\ MN_{2}^{\phi^{+}}\left(G\right) & =\underset{uv\in E\left(G\right)}{\sum }\sigma \left(u\right)\sigma \left(v\right)=683\\ F_{N}^{\phi^{+}}\left(G\right) & =\underset{u\in V\left(G\right)}{\sum }{\left(\sigma \left(u\right)\right)}^{3}=2874\\ MF_{N}^{\phi^{+}}\left(G\right) & =\underset{uv\in E\left(G\right)}{\sum }\left(\sigma {\left(u\right)}^{2}+\sigma {\left(v\right)}^{2}\right)=1521\\ NI^{\phi^{+}}\left(G\right) & =\underset{uv\in E\left(G\right)}{\sum }\frac{\sigma \left(u\right)\sigma \left(v\right)}{\sigma \left(u\right)+\sigma \left(v\right)}=66.604 \end{array}$$

In Figure 4, the values inside the circles represent the proper vertex coloring, while the values outside the circles correspond to the sigma coloring. In sigma coloring, the numbers outside the brackets indicate the initial vertex color, whereas the numbers inside the brackets represent the vertex color sum.

In similar manner, the induced color based topological indices are calculated for UV-4B (N-9-methoxynonyl-1-deoxynojirimycin), ST-148, 4-HPR, Silymarin, Baicalein, Quercetin, Naringenin, Nelfinavir, Ivermectin, Mosnodenvir (JNJ-1802), NITD-688, Metoclopramide, JNJ-A07 and Betulinic acid. The $\phi^{-}$ and $\phi^{+}$ sigma coloring of the isomorphic molecular graphs of potential antivirals of dengue are provided in the Supplementary Material and their computed induced color based topological indices are tabulated in Table 6.

**TABLE 6:** The computed induced color-based topological indices through $\phi^{-}$ and $\phi^{+}$ sigma coloring of 15 potential antivirals of dengue.

Induced color-based topological indices values obtained through $\phi^{-}$ sigma coloring										
Drugs	$ND_{1}^{\phi^{-}}\left(G\right)$	$ND_{2}^{\phi^{-}}\left(G\right)$	$ND_{3}^{\phi^{-}}\left(G\right)$	$ND_{4}^{\phi^{-}}\left(G\right)$	$ND_{5}^{\phi^{-}}\left(G\right)$	$M_{N}^{\phi^{-}}\left(G\right)$	$MN_{2}^{\phi^{-}}\left(G\right)$	$F_{N}^{\phi^{-}}\left(G\right)$	$MF_{N}^{\phi^{-}}\left(G\right)$	$NI^{\phi^{-}}\left(G\right)$
Lycorine	85.139	12.022	1820	10.674	69.933	211	267	739	607	41.081
UV-4B	65.563	11.569	992	10.935	61.667	170	174	512	394	31.671
ST-148	104.986	15.362	1974	13.593	91.083	284	307	996	732	50.242
4-HPR	106.717	15.691	2086	14.455	109.317	297	317	1107	829	49.769
Silymarin	119.140	19.039	2052	17.335	99.417	289	339	905	749	57.969
Baicalein	68.954	10.674	1302	9.829	61.383	291	202	653	487	32.855
Quercetin	76.527	12.780	1278	11.859	64.833	180	214	556	469	37.314
Naringenin	71.948	10.490	1454	9.365	58.5	194	220	674	497	34.791
Nelfinavir	134.575	20.397	2742	18.410	125.4	369	417	1301	1012	65.994
Ivermectin	234.755	33.074	5364	29.951	204.817	616	744	2274	1773	111.724
Mosnodenvir	116.162	18.786	1946	17.616	119.250	288	322	956	798	54.724
NITD-688	111.820	16.679	2114	15.037	103.150	289	329	983	786	53.213
Metoclopramide	60.048	10.119	1004	9.556	55.250	150	167	468	383	28.902
JNJ-A07	122.364	19.139	2108	17.414	110.667	310	347	1000	810	58.655
Betulinic acid	139.370	16.885	4082	19.037	120.267	383	501	1625	1234	65.727

Induced color-based topological indices values obtained through $\phi^{+}$ sigma coloring										
Drugs	$ND_{1}^{\phi^{+}}\left(G\right)$	$ND_{2}^{\phi^{+}}\left(G\right)$	$ND_{3}^{\phi^{+}}\left(G\right)$	$ND_{4}^{\phi^{+}}\left(G\right)$	$ND_{5}^{\phi^{+}}\left(G\right)$	$M_{N}^{\phi^{+}}\left(G\right)$	$MN_{2}^{\phi^{+}}\left(G\right)$	$F_{N}^{\phi^{+}}\left(G\right)$	$MF_{N}^{\phi^{+}}\left(G\right)$	$NI^{\phi^{+}}\left(G\right)$
Lycorine	136.95	9.45	6104	6.37	69.83	532	683	2874	1521	66.6
UV-4B	102.55	9.39	3896	7.11	57.48	389	432	1733	919	50.36
ST-148	160.58	12.57	6912	8.93	84.3	609	726	2937	1571	78.57
4-HPR	160.76	12.81	7210	9.63	109.71	644	726	3426	1787	75.69
Silymarin	196.02	14.83	8892	10.48	99.15	786	912	3936	1967	93.32
Baicalein	108.97	8.55	4848	6.12	55.687	421	500	2061	1081	53.29
Quercetin	127.34	9.86	5810	7.17	68.75	496	592	2460	1290	62.08
Naringenin	103.1	8.22	4374	5.89	54.25	405	462	1963	1020	50.18
Nelfinavir	210.25	17.12	9392	22.08	128.93	819	943	4313	2297	99.88
Ivermectin	365.88	25.81	19752	16.9	256.17	1470	1815	8254	4337	180.07
Mosnodenvir	199.58	14.76	9570	10.17	114.57	809	942	4403	2278	94.95
NITD-688	179.06	13.29	8508	9.38	94.88	706	840	3714	1954	86.05
Metoclopramide	94.64	7.93	4044	6.17	65.72	380	420	1902	995	44.98
JNJ-A07	192.42	15.19	8192	11.01	113.77	772	856	3922	2047	91.82
Betulinic acid	199.29	13.93	11710	10.67	128.21	787	1014	4797	2581	93.17

## 7 QSPR analysis for physicochemical properties of potential antivirals of dengue

The QSPR analysis is carried out between the computed topological indices and physicochemical properties of antiviral drugs for dengue disease, namely, UV-4B (N-9-methoxynonyl-1-deoxynojirimycin), Lycorine, ST-148, 4-HPR, Silymarin, Baicalein, Quercetin, Naringenin, Nelfinavir, Ivermectin, Mosnodenvir (JNJ-1802), NITD-688, Metoclopramide, JNJ-A07 and Betulinic acid. The physicochemical properties of these drugs are tabulated in Table 7 and they were obtained from the database www.chemspider.com. The properties considered for the QSPR analysis include Molar Refraction (MR) $\left(cm^{3}\right)$ , Polarizability (P) $\left(cm^{3}\right)$ , Molar Volume (MV) $\left(cm^{3}\right)$ , Molecular weight (MW) (g/mol) , Heavy Atom Count (HAC) and Complexity(C).

**TABLE 7:** Physicochemical properties of potential antivirals of dengue.

Drugs	MR $\left(cm^{3}\right)$	P $\left(cm^{3}\right)$	MV $\left(cm^{3}\right)$	MW (g/mol)	HAC	C
UV-4B	85.8	34	283	319.44	22	279
Lycorine	74.9	29.7	187	287.31	21	481
ST-148	119.9	47.5	293.2	421.5	29	589
4-HPR	125.1	49.6	361.9	391.5	29	726
Silymarin	120	47.6	315.9	482.4	35	750
Baicalein	69.9	27.7	174.6	270.24	20	413
Quercetin	73.3	29.1	168	302.23	22	488
Naringenin	70.3	27.9	183.3	272.25	20	363
Nelfinavir	162.4	64.4	463.1	567.8	40	830
Ivermectin	230.7	91.5	708.4	875.1	62	1680
Mosnodenvir	138.4	54.9	400.8	583	39	951
NITD-688	135	53.5	373.2	500.7	34	880
Metoclopramide	79.7	31.6	252.3	299.79	20	300
JNJ-A07	141.1	55.9	410.6	579	40	843
Betulinic acid	133.2	52.8	428.8	456.7	33	861

**FIGURE 5:**
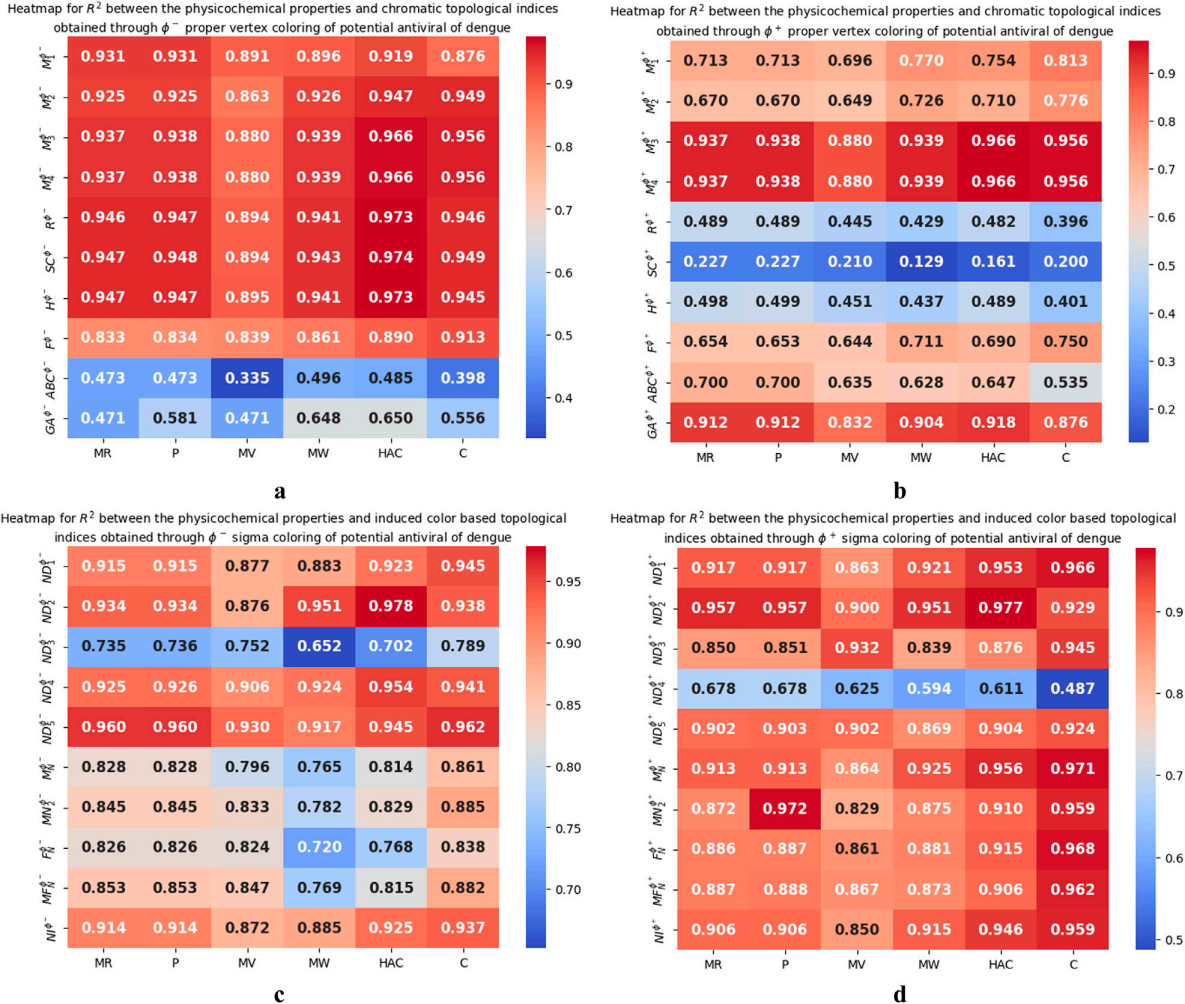
**(a)** Heatmap of $R^{2}$ between the physicochemical properties and $\phi^{-}$ chromatic topological indices **(b)** Heatmap of $R^{2}$ between the physicochemical properties and $\phi^{+}$ chromatic topological indices **(c)** Heatmap of $R^{2}$ between the physicochemical properties and $\phi^{-}$ induced color-based topological indices **(d)** Heatmap of $R^{2}$ between the physicochemical properties and $\phi^{+}$ induced color-based topological indices.

The linear regression Y=A+B(X) is used in QSPR analysis to explore the physicochemical properties of the aforementioned drugs. Here, Y represents the physicochemical property, X denotes the computed topological index and A and B are constants. The statistical parameters include $R^{2}$ (coefficient of determination), R (correlation coefficient), F (F-statistics) and SE (standard error), which collectively evaluate the model’s performance. Specifically, $R^{2}$ measures the proportion of the variance in the dependent variable explained by the regression model, R indicates the strength and direction of the linear relationship between the predicted and actual value, F tests the overall significance of the regression model and SE quantifies the average deviation of the observed values from the predicted value. The QSPR graph model maximizes $R^{2},R$ and F values and minimizes SE value in the statistical analysis. The squared correlation coefficient values $\left(R^{2}\right)$ determined between the indices and physicochemical properties of potential antiviral drugs are presented in the form of heatmaps and are shown in Figure 5. The best-fitting and most predictable linear regression equations, having the maximum $R^{2}$ values, are summarized in Table 8. It is noted that the physicochemical properties hold great significance since R>0.8 and the p-value is less than 0.05. Compared to other regression models, linear regression analysis demonstrates significant outcomes, with a high coefficient value and a smaller standard error.

**TABLE 8:** The statistical parameters for the highly correlated chromatic topological indices and induced color-based topological indices.

The statistical parameters for the highly correlated chromatic topological indices							
Property	Type	Linear regression equation	$R^{2}$	R	F	S.E	p-value
MR	$\phi^{-}$	$MR=0.7+5.31\left(SC^{\phi^{-}}\right)$	0.947	0.973	233.538	10.492	0.000
		$MR=0.23+4.65\left(H^{\phi^{-}}\right)$	0.947	0.973	230.360	10.561	0.000
	$\phi^{+}$	$MR=5.52+2.84\left(M_{3}^{\phi^{+}}\right)$	0.937	0.968	194.217	11.445	0.000
		$MR=5.52+5.67\left(M_{4}^{\phi^{+}}\right)$					
P	$\phi^{-}$	$P=0.26+2.1\left(SC^{\phi^{-}}\right)$	0.948	0.973	235.4	4.145	0.000
	$\phi^{+}$	$P=2.18+1.13\left(M_{3}^{\phi^{+}}\right)$	0.938	0.968	195.213	4.527	0.000
		$P=2.18+2.25\left(M_{4}^{\phi^{+}}\right)$					
MV	$\phi^{-}$	$MV=36.58+14.69\left(H^{\phi^{-}}\right)$	0.895	0.946	110.54	48.201	0.000
	$\phi^{+}$	$MV=18.72+8.94\left(M_{3}^{\phi^{+}}\right)$	0.880	0.938	95.587	51.413	0.000
		$MV=18.72+17.88\left(M_{4}^{\phi^{+}}\right)$					
MW	$\phi^{-}$	$MW=1.14+19.99\left(SC^{\phi^{-}}\right)$	0.943	0.971	213.911	41.315	0.000
	$\phi^{+}$	$MW=17.9+10.73\left(M_{3}^{\phi^{+}}\right)$	0.939	0.969	199.886	42.654	0.000
		$MW=18.72+17.88\left(M_{4}^{\phi^{+}}\right)$					
HAC	$\phi^{-}$	$HAC=0.21+1.4\left(SC^{\phi^{-}}\right)$	0.974	0.987	479.379	1.938	0.000
	$\phi^{+}$	$HAC=1.44+0.75\left(M_{3}^{\phi^{+}}\right)$	0.966	0.983	369.757	2.198	0.000
		$HAC=1.44+1.5\left(M_{4}^{\phi^{+}}\right)$					
C	$\phi^{-}$	$C=0.0214+23.08\left(M_{3}^{\phi^{-}}\right)$	0.956	0.978	283.785	77.016	0.000
		$C=0.0214+46.16\left(M_{4}^{\phi^{-}}\right)$					
	$\phi^{+}$	$C=-0.0214+23.08\left(M_{3}^{\phi^{+}}\right)$					
		$C=0.021+46.16\left(M_{4}^{\phi^{+}}\right)$					

The statistical parameters for the highly correlated induced color-based topological indices							
MR	$\phi^{-}$	$MR=10.27+1.1\left(ND_{5}^{\phi^{-}}\right)$	0.960	0.980	312.777	9.128	0.000
	$\phi^{+}$	$MR=3.37+9.35\left(ND_{2}^{\phi^{+}}\right)$	0.957	0.978	290.070	9.463	0.000
P	$\phi^{-}$	$P=4.06+0.44\left(ND_{5}^{\phi^{-}}\right)$	0.960	0.980	315.138	3.606	0.000
	$\phi^{+}$	$P=1.35+3.71\left(ND_{2}^{\phi^{+}}\right)$	0.957	0.978	292.482	3.738	0.000
MV	$\phi^{-}$	$MV=8.98+3.53\left(ND_{5}^{\phi^{-}}\right)$	0.930	0.964	172.404	39.346	0.000
	$\phi^{+}$	$MV=64.07+2.69\left(ND_{5}^{\phi^{+}}\right)$	0.902	0.950	120.195	46.421	0.000
MW	$\phi^{-}$	$MW=8.63+27.76\left(ND_{2}^{\phi^{-}}\right)$	0.951	0.975	255.039	38.013	0.000
	$\phi^{+}$	$MW=13.8+35.19\left(ND_{2}^{\phi^{+}}\right)$	0.951	0.975	251.848	38.241	0.000
HAC	$\phi^{-}$	$HAC=0.39+1.93\left(ND_{2}^{\phi^{-}}\right)$	0.978	0.989	567.116	1.7852	0.000
	$\phi^{+}$	$HAC=-0.76+2.46\left(ND_{2}^{\phi^{+}}\right)$	0.977	0.989	562.695	1.792	0.000
C	$\phi^{-}$	$C=0.0167+8.9\left(ND_{5}^{\phi^{-}}\right)$	0.962	0.981	330.749	71.562	0.000
	$\phi^{+}$	$C=47.48+0.21\left(F_{N}^{\phi^{+}}\right)$	0.968	0.984	389.915	66.099	0.000

**FIGURE 6:**
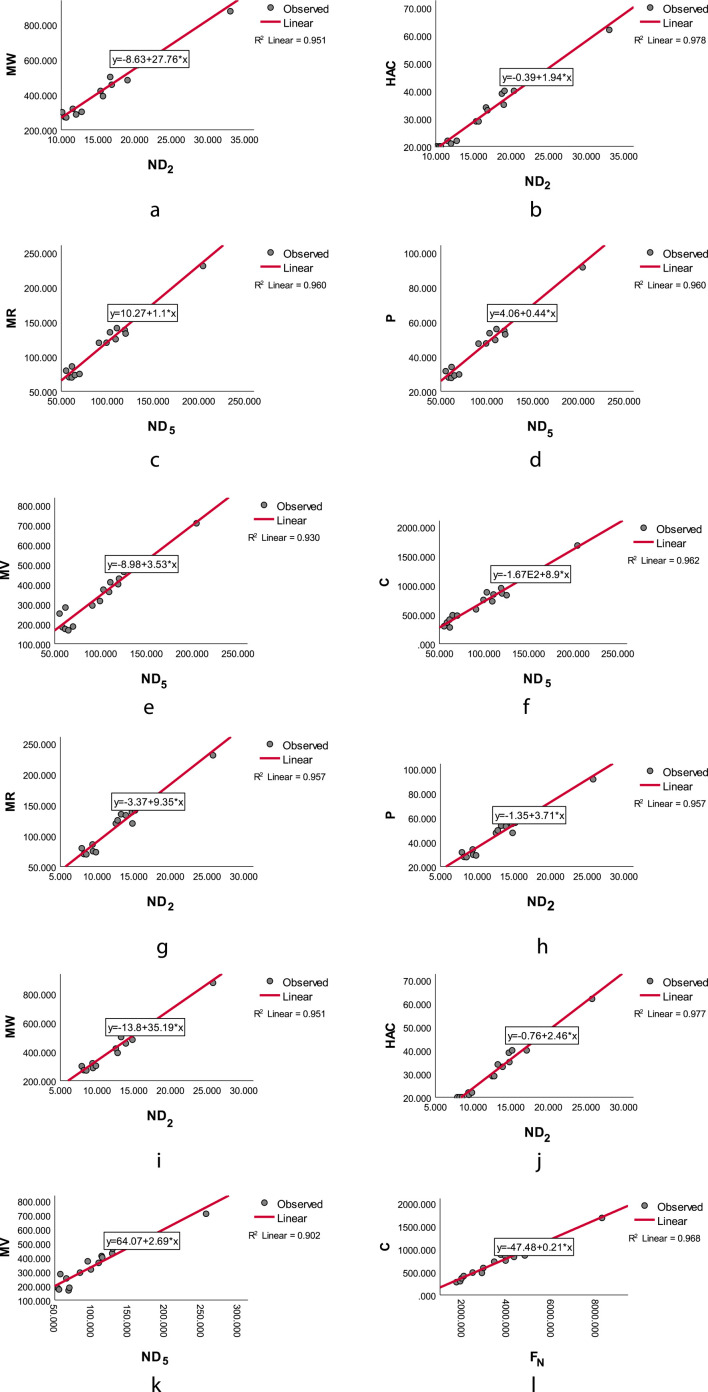
The linear regression curve of **(a)** $\phi^{-}$ second induced color index $\left(ND_{2}^{\phi^{-}}\right)$ with Molar Weight (MW) **(b)** $\phi^{-}$ second induced color index $\left(ND_{2}^{\phi^{-}}\right)$ with Heavy Atom Count(HAC) **(c)** $\phi^{-}$ fifth induced color index $\left(ND_{5}^{\phi^{-}}\right)$ with Molar Refraction(MR) **(d)**+ $\phi^{-}$ fifth induced color index $\left(ND_{5}^{\phi^{-}}\right)$ with Polarizability(P) **(e)** $\phi^{-}$ fifth induced color index $\left(ND_{5}^{\phi^{-}}\right)$ with Molar Volume (MV) **(f)** $\phi^{-}$ fifth induced color index $\left(ND_{5}^{\phi^{-}}\right)$ with Complexity (C) **(g)** $\phi^{+}$ second induced color index $\left(ND_{2}^{\phi^{+}}\right)$ with Molar Refraction (MR) **(h)** $\phi^{+}$ second induced color index $\left(ND_{2}^{\phi^{+}}\right)$ with Polarizability (P) **(i)** $\phi^{+}$ second induced color index $\left(ND_{2}^{\phi^{+}}\right)$ with Molar Weight(MW) **(j)** $\phi^{+}$ second induced color index $\left(ND_{2}^{\phi^{+}}\right)$ with Heavy Atom Count(HAC) **(k)** $\phi^{+}$ fifth induced color index $\left(ND_{5}^{\phi^{+}}\right)$ and $\phi^{+}$ Forgotten induced color index $\left(F_{N}^{\phi^{+}}\right)$ with Molar Volume (MV) **(l)** $\phi^{+}$ fifth induced color index $\left(ND_{5}^{\phi^{+}}\right)$ and $\phi^{+}$ Forgotten induced color index $\left(F_{N}^{\phi^{+}}\right)$ with Complexity (C).

The $R^{2}$ values obtained through chromatic topological indices and induced color-based indices are compared and analyzed. The results show that the induced color-based indices significantly outperform chromatic topological indices. The curve fits between the computed induced color-based topological indices and drug properties, obtained through linear regression with the highest $R^{2}$ values and p<0.05 , are illustrated in Figure 6.

## 8 Y-randomization test

The Y-randomization test (also known as response permutation test) is performed to ensure that the developed QSPR analysis is not influenced by chance correlations. This test is a crucial validation technique in QSPR analysis to assess whether the observed relationship between the physicochemical properties and the computed topological indices are statistically significant.

In this procedure, the dependent variable (Y-values), representing the physicochemical properties, is randomly shuffled, while the independent variables (X-values), representing the topological indices, remain unchanged. A linear regression is then trained on the randomized dataset, and its coefficient of determination $\left(R^{2}\right)$ is compared with that of the original model. A significant drop in $R^{2}$ value after randomization indicates that the original model captures meaningful structure-property relationships.

The original and scrambled $R^{2}$ values and the original and scrambled mean squared error (MSE) for the proposed QSPR analysis are summarized in Table 9.

**TABLE 9:** Comparison of original and scrambled R^2^ and mean squared error value.

Chromatic topological indices					
Properties	Indices	Original $R^{2}$	Original MSE	Mean scrambled $R^{2}$	Mean scrambled MSE
MR	$SC^{\phi^{-}}$	0.9473	95.4128	0.0738	1675.854
P	$SC^{\phi^{-}}$	0.9477	14.8893	0.0738	263.4953
MW	$SC^{\phi^{-}}$	0.9427	1479.3035	0.0729	23938.322
HAC	$SC^{\phi^{-}}$	0.9736	3.2544	0.0728	114.2873
MR	$H^{\phi^{-}}$	0.9466	96.6586	0.0739	1675.7782
MV	$H^{\phi^{-}}$	0.8948	2013.5507	0.0761	17677.9033
C	$M_{3}^{\phi^{-}}$	0.9562	5140.5841	0.0717	108944.268
C	$M_{4}^{\phi^{-}}$	0.9562	5140.5841	0.0717	108944.268
MR	$M_{3}^{\phi^{+}}$ , $M_{4}^{\phi^{+}}$	0.9373	113.518	0.0735	1676.4252
P	$M_{3}^{\phi^{+}}$ , $M_{4}^{\phi^{+}}$	0.9376	17.7631	0.0735	263.5855
MV	$M_{3}^{\phi^{+}}$ , $M_{4}^{\phi^{+}}$	0.8803	2290.8241	0.0761	17679.3411
MW	$M_{3}^{\phi^{+}}$ , $M_{4}^{\phi^{+}}$	0.9389	1576.7638	0.0725	23948.493
HAC	$M_{3}^{\phi^{+}}$ , $M_{4}^{\phi^{+}}$	0.966	4.1865	0.0724	114.3431
C	$M_{3}^{\phi^{+}}$ , $M_{4}^{\phi^{+}}$	0.9562	5140.5841	0.0717	108944.268

Induced color-based topological indices					
MW	$ND_{2}^{\phi^{-}}$	0.9515	1252.3192	0.0728	23941.1061
HAC	$ND_{2}^{\phi^{-}}$	0.9776	2.7622	0.0727	114.2989
MR	$ND_{5}^{\phi^{-}}$	0.9601	72.2054	0.0743	1675.0849
P	$ND_{5}^{\phi^{-}}$	0.9604	11.2712	0.0743	263.3742
C	$ND_{5}^{\phi^{-}}$	0.9622	4438.2593	0.0728	108814.8517
MV	$ND_{5}^{\phi^{-}}$	0.9299	1341.6891	0.0766	17668.6861
MR	$ND_{2}^{\phi^{+}}$	0.9571	77.6152	0.074	1675.4974
P	$ND_{2}^{\phi^{+}}$	0.9574	12.1071	0.074	263.4393
MW	$ND_{2}^{\phi^{+}}$	0.9509	1267.4084	0.073	23935.4336
HAC	$ND_{2}^{\phi^{+}}$	0.9774	2.7834	0.0729	114.2729
MV	$ND_{5}^{\phi^{+}}$	0.9024	1867.5851	0.0767	17666.3363
C	$F_{N}^{\phi^{+}}$	0.9677	3786.5248	0.0718	108931.9603

### 8.1 Inference from Y-randomization test

(i) The original R^2^; values for the physicochemical properties of potential antivirals of dengue disease are consistently high, with most values close to or exceeding 0.8. This indicates that the regression models effectively explain a significant proportion of variance in the observed data. Additionally, the original MSE values are low, demonstrating the accuracy of the predictions.
(ii) The mean scrambled R^2^; values remain consistently low (approximately 0.07 across all properties), suggesting a weak or nonexistent relationship between the scrambled observed values and the predicted values. Furthermore, the mean scrambled MSE values are substantially higher than the original MSE values, confirming that randomization disrupts predictive accuracy.
(iii) The substantial difference between the original and scrambled R^2^; values, along with the significant difference in MSE values, underscores that the predictive performance of the analysis is not attributable to random chance.

These results affirm that the regression models effectively capture meaningful relationships between the computed topological indices and the physicochemical properties of the considered dengue-treating drugs. Consequently, the Y-randomization test validates the statistical significance and robustness of the QSPR linear regression analysis.

## 9 Results and discussion

The chromatic topological indices that yield the highest correlation in the QSPR analysis for the physicochemical properties of UV-4B (N-9-methoxynonyl-1-deoxynojirimycin), Lycorine, ST-148, 4-HPR, Silymarin, Baicalein, Quercetin, Naringenin, Nelfinavir, Ivermectin, Mosnodenvir (JNJ-1802), NITD-688, Metoclopramide, JNJ-A07 and Betulinic acid are as follows:

(i) The $\phi^{-}$ chromatic sum connectivity index $\left(SC^{\phi^{-}}\right)$ shows an excellent corrlation coefficient of Molar Refraction(MR), Polarizability (P), Molar weight(MW) and Heavy Atom Count(HAC).
(ii) The $\phi^{-}$ chromatic harmonic index $\left(H^{\phi^{-}}\right)$ is the best suitable index to predict Molar Refraction(MR) and Molar Volume(MV).
(iii) The $\phi^{-}$ chromatic irregularity index $\left(M_{3}^{\phi^{-}}\right)$ and $\phi^{-}$ chromatic total irregularity index $\left(M_{4}^{\phi^{-}}\right)$ are the best indices for the prediction of Complexity (C).
(iv) The $\phi^{+}$ chromatic irregularity index $\left(M_{3}^{\phi^{+}}\right)$ and $\phi^{+}$ chromatic total irregularity index $\left(M_{4}^{\phi^{+}}\right)$ are the best suited indices for predicting Molar Refraction (MR), Polarizability (P), Molar Volume (MV), Molar Weight(MW), Heavy Atom Count(HAC) and Complexity (C).

The induced color-based topological indices that yield the highest correlation the QSPR analysis for the physicochemical properties of UV-4B (N-9-methoxynonyl-1-deoxynojirimycin), Lycorine, ST-148, 4-HPR, Silymarin, Baicalein, Quercetin, Naringenin, Nelfinavir, Ivermectin, Mosnodenvir (JNJ-1802), NITD-688, Metoclopramide, JNJ-A07 and Betulinic acid are as follows:

(i) The $\phi^{-}$ second induced color index $\left(ND_{2}^{\phi^{-}}\right)$ is highly correlated with the properties, Molar Weight(MW) and Heavy Atom Count(HAC).
(ii) The $\phi^{-}$ fifth induced color index $\left(ND_{5}^{\phi^{-}}\right)$ is the best suited index to predict Molar Refraction (MR), Polarizability(P), Complexity (C) and Molar Volume(MV).
(iii) The $\phi^{+}$ second induced color index $\left(ND_{2}^{\phi^{-}}\right)$ is the best suited index to predict Molar Refraction (MR), Polarizability(P), Molar Weight(MW) and Heavy Atom Count(HAC).
(iv) The $\phi^{+}$ fifth induced color index $\left(ND_{5}^{\phi^{+}}\right)$ is the best one for the prediction of the property Molar Volume(MV).
(v) The $\phi^{+}$ forgotten induced color index $F_{N}^{\phi^{+}}$ is highly correlated with the property Complexity (C).

### 9.1 Comparison of chromatic topological indices and induced color-based topological indices

The predictive capabilities of the newly introduced chromatic topological indices and induced color-based topological indices are compared to analyze their ability to model physicochemical properties. Notably, the induced color-based topological indices demonstrated consistently higher correlations, with $R^{2}$ values mostly exceeding 0.8, indicating more robust relationships with physicochemical properties. In contrast, chromatic topological indices exhibited greater variability in correlation strengths, with $R^{2}$ values ranging from 0.3 to 0.9. This significant difference suggests that the induced color-based indices can serve as more reliable predictors of physicochemical properties of chemical molecule. Additionally, from Figure 5, it is observed that the induced color-based indices consistently showed strong correlations across various property combinations, while chromatic indices displayed greater fluctuations in correlation strengths. The minimum $R^{2}$ values for induced color-based indices remained relatively high, ranging from 0.7 to 0.8, compared to the minimum $R^{2}$ values for chromatic indices, which ranged from 0.3 to 0.4. This difference in minimum $R^{2}$ values further emphasizes the strong predictive capability of induced color-based indices. These findings of QSPR analysis indicate that the induced color-based topological indices serve as an effective tool for modeling and predicting the physicochemical properties in chemical molecule.

## 10 Conclusion

Ten novel induced color-based topological indices and six chromatic-based topological indices were introduced to analyze the molecular structures of the antiviral drugs of dengue. The induced color-based indices were computed through sigma coloring, while chromatic topological indices were derived from proper vertex coloring. The QSPR analysis was performed between the physicochemical properties of dengue treating drugs and the computed topological indices of their molecular structures of the drugs. The results showed that the specific induced color-based indices such as the second induced color index $\left(ND_{2}^{\phi_{t}}\right)$ , fifth induced color index $\left(ND_{5}^{\phi_{t}}\right)$ and forgotten induced color index $\left(F_{N}^{\phi_{t}}\right)$ exhibited strong correlations with the properties. A comparative analysis between chromatic index and induced color-based index indicated that the induced color-based indices offer stronger correlations, suggesting their superior ability to capture structural features relevant to drug properties. Furthermore, the Y-randomization test confirmed that the QSPR analysis was not influenced by chance correlation. The findings of our work established the induced color-based indices as reliable predictors of the physicochemical properties of dengue antiviral drugs. The analysis using the color-based topological indices provide a foundation for further exploration in drug design and molecular property prediction.

## 11 Future work

(i) QSPR graph model proposed in this article can be extended to other chemical molecules and drugs to explore their properties
(ii) The Quantitative Structure-Activity Relationship(QSAR) and Quantitative Structure-Toxicity Relationship(QSTR) analysis can be performed between the computed topological indices and other properties through the induced color-based topological indices and chromatic topological indices for the potential antivirals of dengue.
(iii) The analysis of isomorphic molecular graph of chemical molecules through color based topological indices will provide us with the best model to predict the properties of the molecules.

## Data Availability

The original contributions presented in the study are included in the article/Supplementary Material, further inquiries can be directed to the corresponding author.
