# Supplementary material for: QSPR graph model to explore physicochemical properties of potential antiviral drugs of dengue disease through novel coloring-based topological indices
Source: Front Chem. 2025 Aug 18;13:1599715. doi: 10.3389/fchem.2025.1599715 (PMC12400515; doi:10.3389/fchem.2025.1599715)
Supplement: Supplementary file 1 [file DataSheet1.pdf]

Supplementary Material

1 SUPPLEMENTARY TABLES AND FIGURES

1.1 Figures

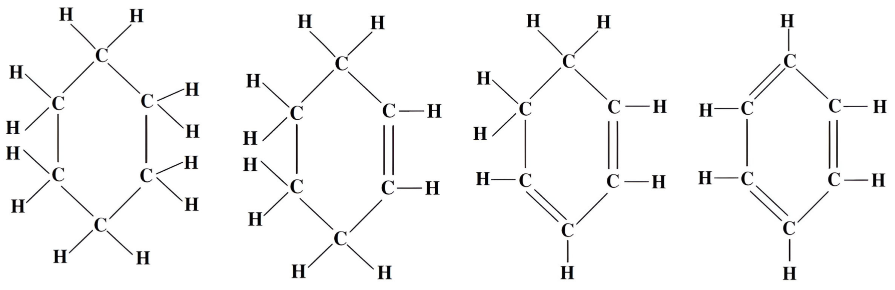

(A) (B) (C) (D)

Figure S1: (A) Chemical structure of Cyclohexane (B) Chemical structure of Cyclohexene (C) Chemical structure of Cyclohexadiene (D) Chemical structure of benzene.

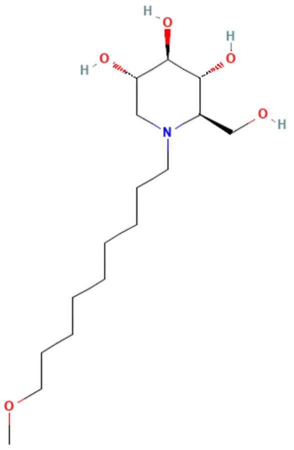

Figure 2a

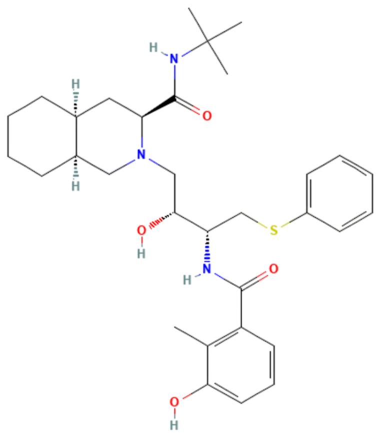

Figure 2b

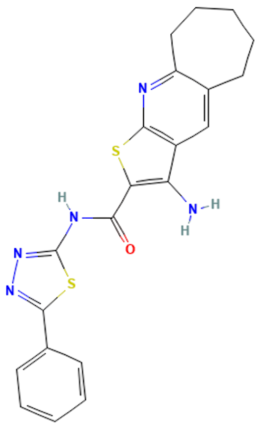

Figure 2c

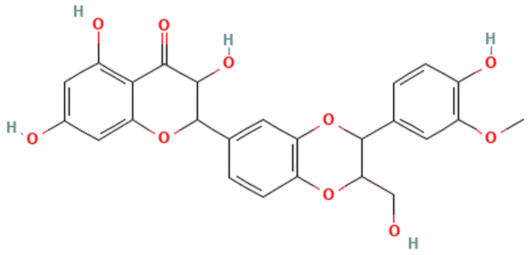

Figure 2d

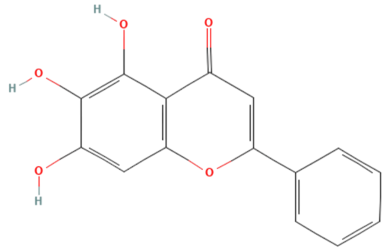

Figure 2e

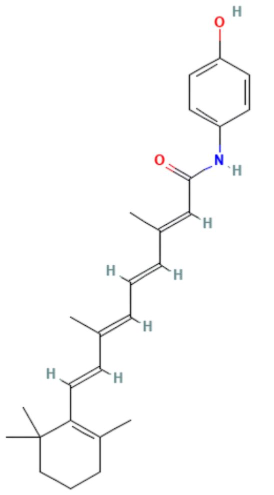

Figure 2f

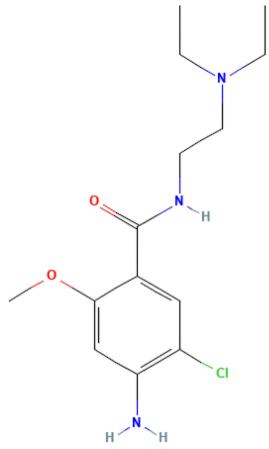

Figure 2g

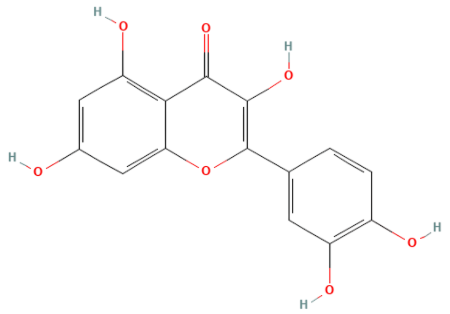

Figure 2h

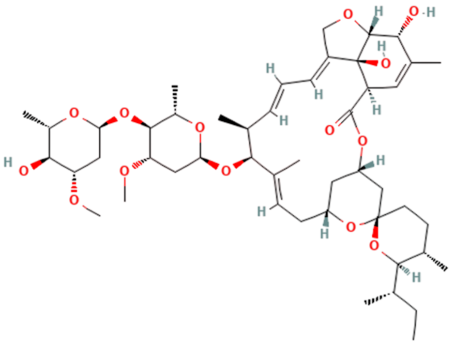

Figure 2i

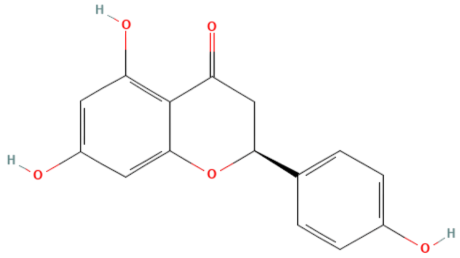

Figure 2j

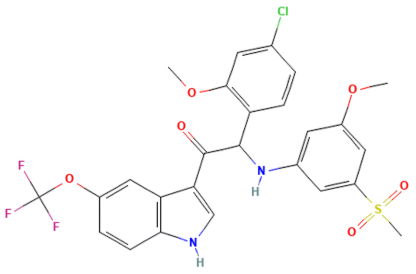

Figure 2k

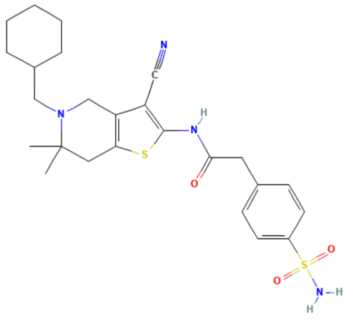

Figure 2l

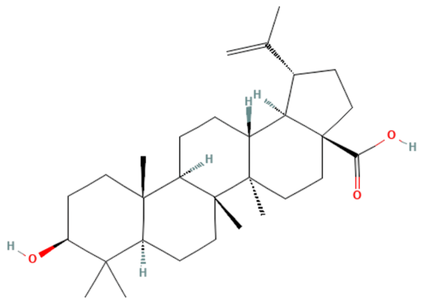

Figure 2m

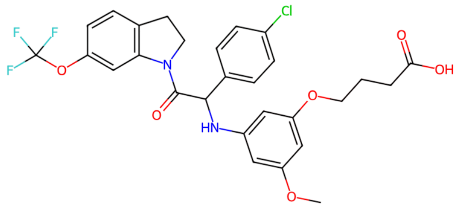

Figure 2n

Figure 2: The chemical structure of (A)UV-4B (B)Nelfinavir (C)ST-148 (D) Silymarin (E)Baicalein (F)4-HPR (G)Metoclopramide (H)Quercetin (I)Ivermectin (J)Naringenin (K)Mosnodenvir (L)NITD-688 (M)Beulinic acid (N)JNJ-A07

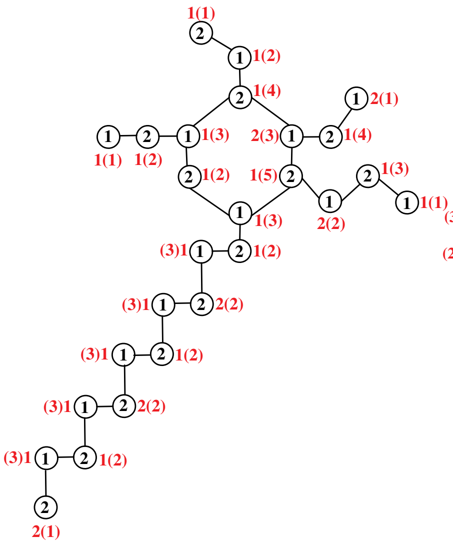

Figure 3a

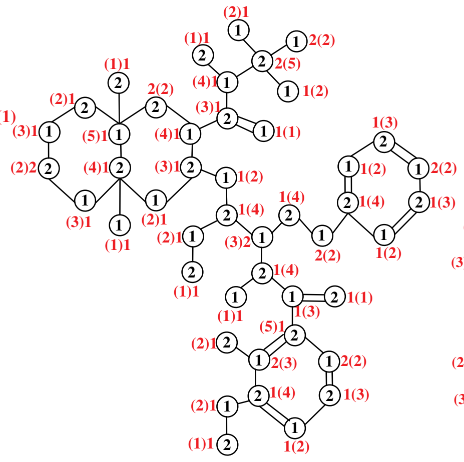

Figure 3b

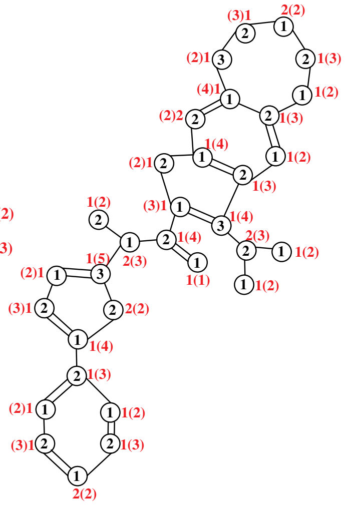

Figure 3c

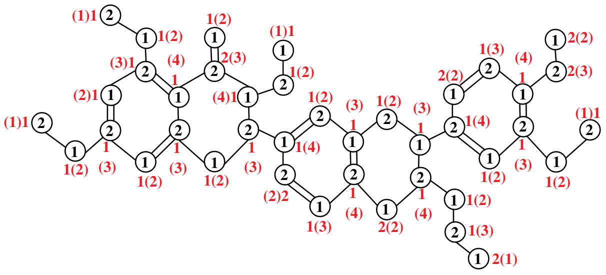

Figure 3d

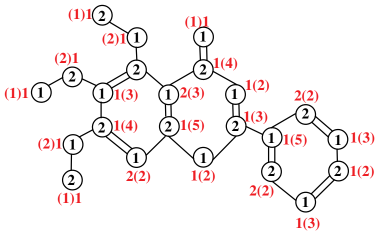

Figure 3e

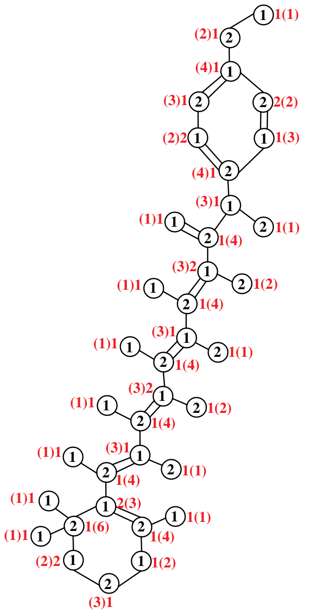

Figure 3f

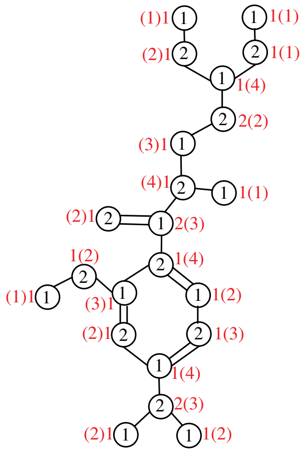

Figure 3g

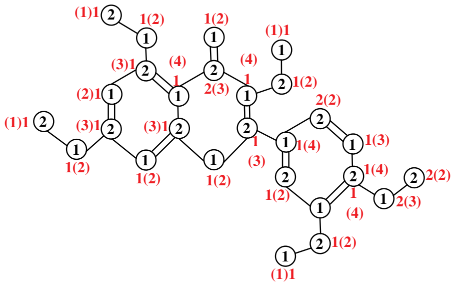

Figure 3h

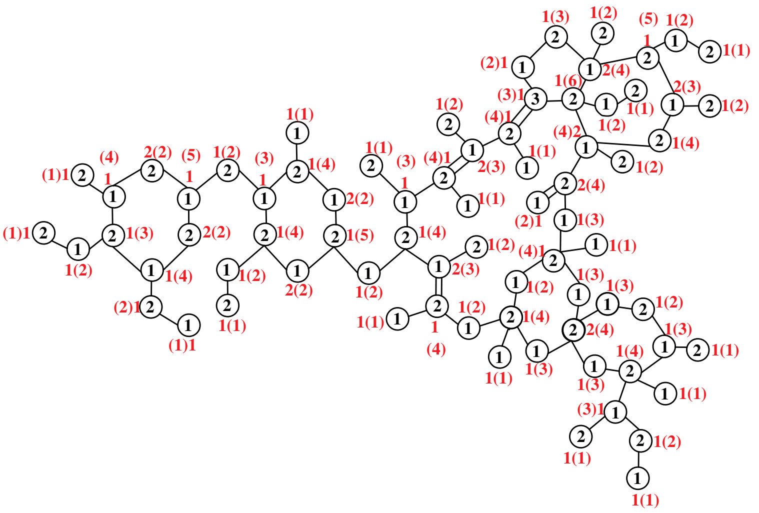

Figure 3i

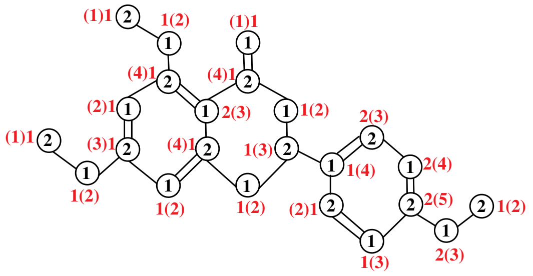

Figure 3j

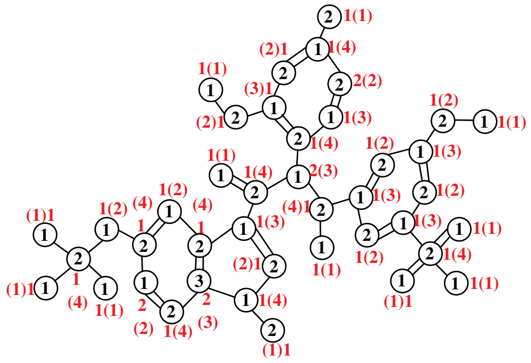

Figure 3k

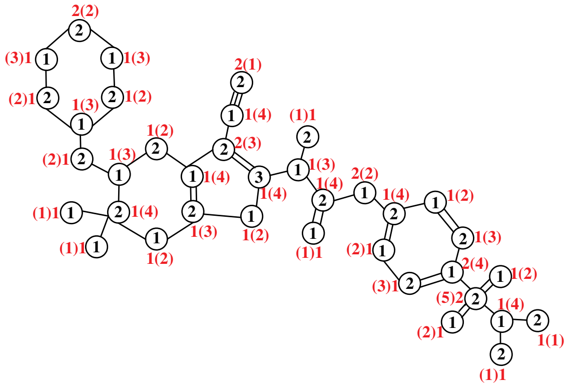

Figure 3l

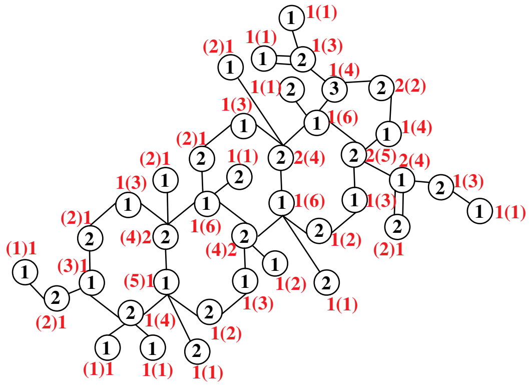

Figure 3m

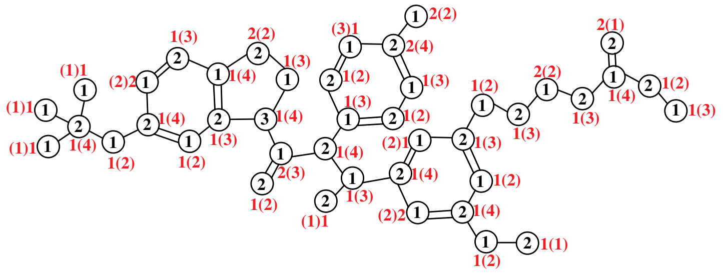

Figure 3n

Figure 3o: ϕ^- proper vertex coloring and ϕ^- sigma coloring of the isomorphic molecular graph of (A)UV-4B (B)Nelfinavir (C)ST-148 (D) Silymarin (E)Baicalein (F)4-HPR (G)Metoclopramide (H)Quercetin (I)Ivermectin (J)Naringenin (K)Mosnodenvir (L)NITD-688 (M)Beulinic acid (N)JNJ-A07

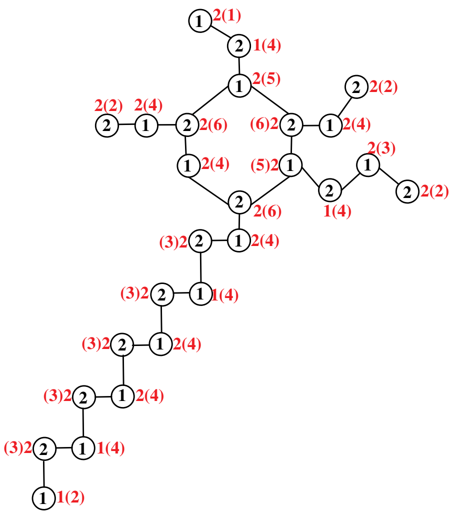

Figure 3a

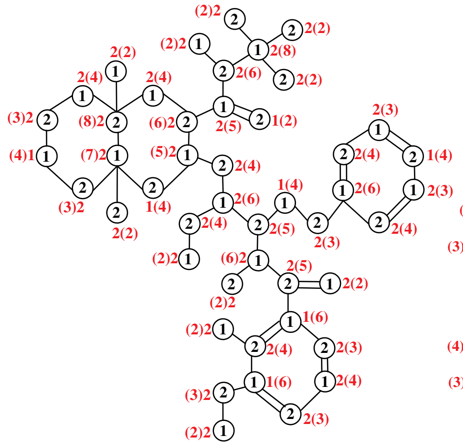

Figure 3b

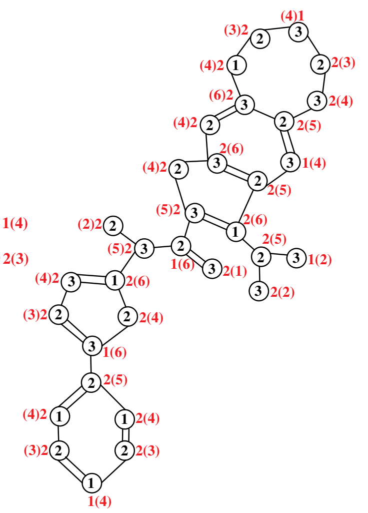

Figure 3c

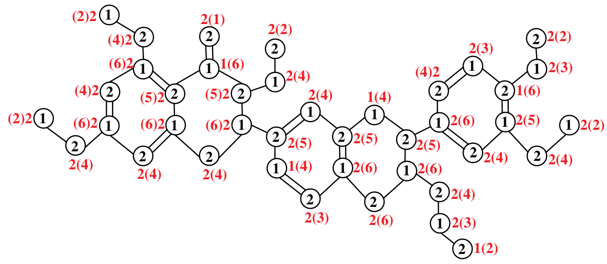

Figure 3d

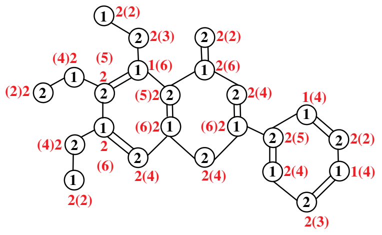

Figure 3e

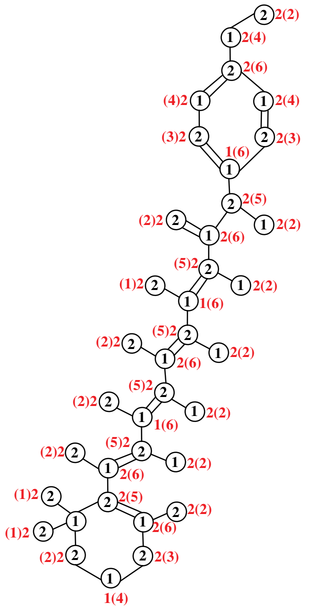

Figure 3f

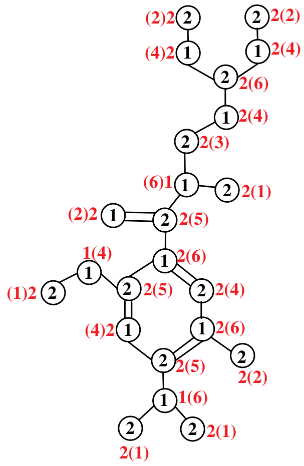

Figure 3g

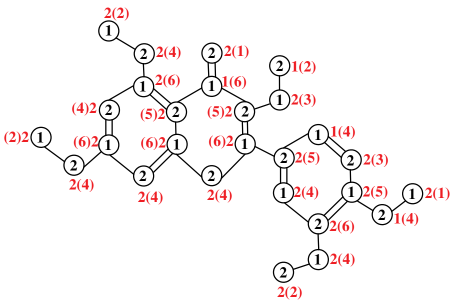

Figure 3h

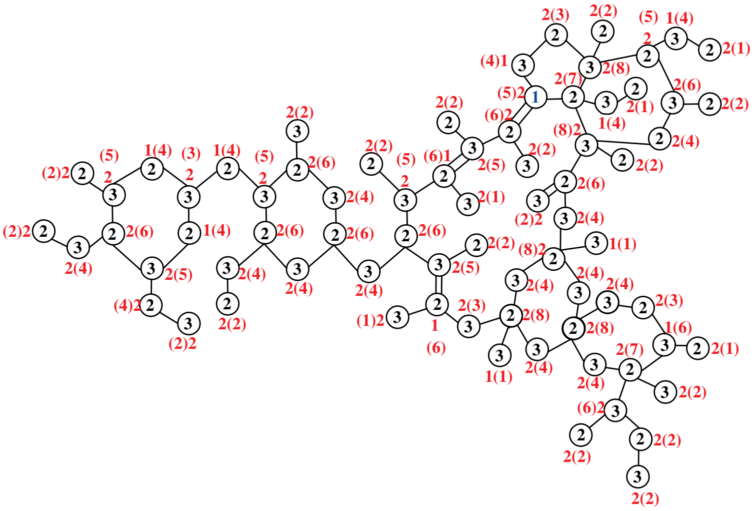

Figure 3i

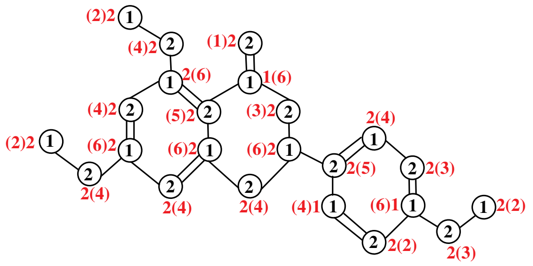

Figure 3j

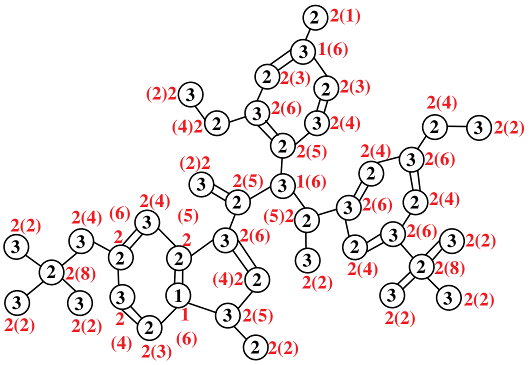

Figure 3k

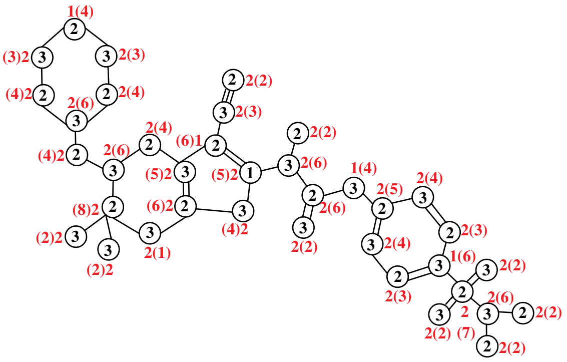

Figure 3l

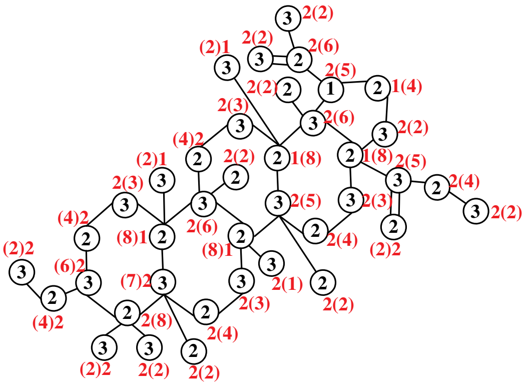

Figure 3m

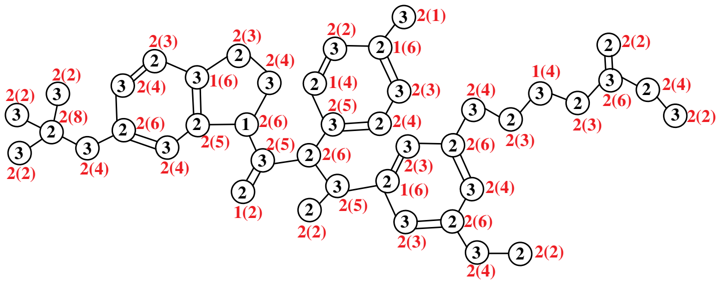

Figure 3n

Figure 3: ϕ^+ proper vertex coloring and ϕ^+ sigma coloring of the isomorphic molecular graph of (A)UV-4B (B)Nelfinavir (C)ST-148 (D) Silymarin (E)Baicalein (F)4-HPR (G)Metoclopramide (H)Quercetin (I)Ivermectin (J)Naringenin (K)Mosnodenvir (L)NITD-688 (M)Beulinic acid (N)JNJ-A07
